# Corals reveal ENSO-driven synchrony of climate impacts on both terrestrial and marine ecosystems in northern Borneo

**DOI:** 10.1038/s41598-020-60525-1

**Published:** 2020-02-28

**Authors:** Hedwig Krawczyk, Jens Zinke, Nicola Browne, Ulrich Struck, Jennifer McIlwain, Michael O’Leary, Dieter Garbe-Schönberg

**Affiliations:** 1 School of Geography, Geology and the Environment, University of Leicester, University Rd, LE1 7RH Leicester, United Kingdom; 2 Molecular and Life Sciences, Curtin University, Kent St, Bentley WA, 6102 Perth, Australia; 3 Curtin Malaysia Research Institute, Curtin University Malaysia, CDT 250, 98009 Miri, Sarawak Malaysia; 4 Australian Institute of Marine Science, PMB No.3, 4810 Townsville, Australia; 5 Museum für Naturkunde, Leibniz Institute for Evolution and Biodiversity Science, Invalidenstraße 43, 10115 Berlin, Germany; 6 Institute for Geosciences, Paleontology, Freie Universität Berlin, Malteserstraße 74-100, 12249 Berlin, Germany; 7 School of Earth Sciences, The University of Western Australia, 35 Stirling Highway, 6009 Perth, Australia; 8 Institute for Geosciences, Christian-Albrechts-Universität zu Kiel, Ludewig-Meyn-Str. 10, 24118 Kiel, Germany

**Keywords:** Climate change, Palaeoceanography, Palaeoclimate, Environmental impact

## Abstract

Extreme climate events, such as the El Niños in 1997/1998 and 2015/16, have led to considerable forest loss in the Southeast Asian region following unprecedented drought and wildfires. In Borneo, the effects of extreme climate events have been exacerbated by rapid urbanization, accelerated deforestation and soil erosion since the 1980s. However, studies quantifying the impact of interannual and long-term (>3 decades) climatic and anthropogenic change affecting Borneo’s coastal and coral reef environments are lacking. Here, we used coral cores collected in Miri-Sibuti Coral Reefs National Park, Sarawak (Malaysia) to reconstruct the spatio-temporal dynamics of sea surface temperature and oxygen isotopic composition of seawater from 1982 to 2016, based on paired oxygen isotope and Sr/Ca measurements. The results revealed rising sea surface temperatures of 0.26 ± 0.04 °C per decade since 1982. Reconstructed δ^18^O_sw_ displayed positive excursion during major El Niño events of 1983, 1997/98 and 2015/16, indicating drought conditions with less river runoff, rainfall and higher ocean salinities. La Niñas were generally associated with lower δ^18^O_sw_. We observed a long-term shift from more saline conditions between 1982 and 1995 towards less saline conditions after 1995, which are in agreement with the regional freshening trend, punctuated by saline excursion during El Niños. The decadal shifts were found to be driven by the Pacific Decadal Oscillation (PDO). This study provides the first long-term data on El Niño Southern Oscillation (ENSO)-driven synchrony of climate impacts on both terrestrial and marine ecosystems in northern Borneo. Our results suggest that coral records from northern Borneo are invaluable archives to detect regional ENSO and PDO impacts, and their interaction with the Asian-Australian monsoon, on the hydrological balance in the southern South China Sea beyond the past three decades.

## Introduction

Southeast Asia (SEA) harbours an astonishing diversity of stony corals, comprising approximately 28% of the global total, located in the Maritime Continent^1,2^. Local ecological threats, such as coastal development, overfishing/destructive fishing and pollution, detrimentally impact 95% of the coral reefs in SEA and are amplified by large-scale global pressures^2^. Over the past 40 years, the Maritime Continent has undergone intense economic development and urbanization, drastically increasing human-induced pressures on the marine coastal ecosystems^3–6^. In Borneo, rapid urbanization of coastal zones and expansion of palm oil plantations has resulted in some of the highest levels of deforestation among all humid tropical regions of the world^7–10^. Approximately 26% of old-growth rainforest has been lost since the early 1970s^7–10^. Anthropogenic land-use change can severely impact the erosion potential of the hinterland soils, as well as the freshwater and sediment discharge into the nearshore environment, which in turn alters water quality on coral reefs^11,12^.

**Figure 1:**
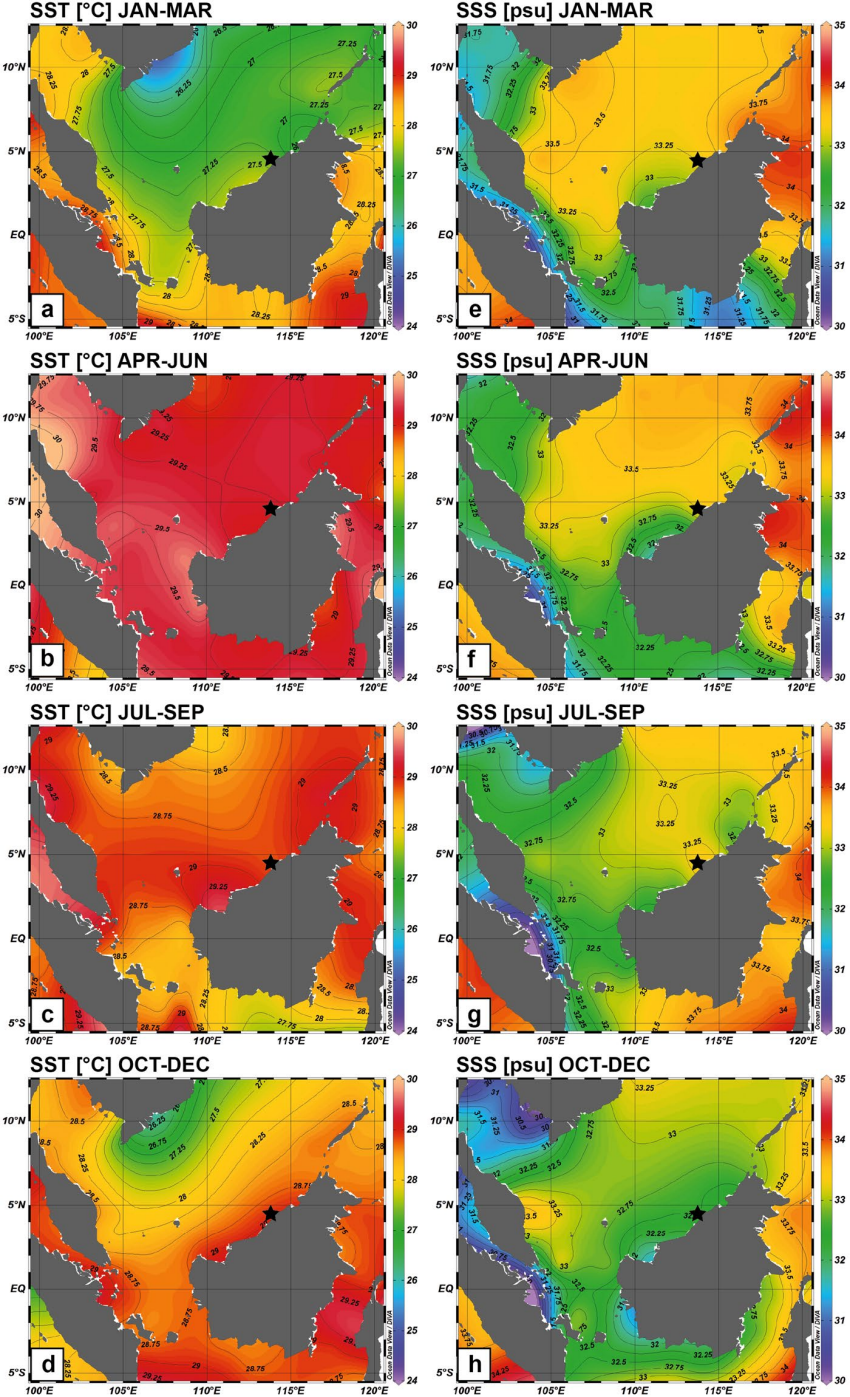
Seasonal SST (**a**–**d**) and salinity (SSS; **e**–**h**) in the South China Sea. Study area is marked with black star. All plots were made with Ocean Data View^86^ using the World Ocean Atlas 2009 data^87,88^.

In northern Borneo, natural climatic events paired with currently unquantified contributions from climate change may exacerbate the regional anthropogenic impacts on coral reefs. The regional climate is strongly influenced by the Asian-Australian monsoon system^13–15^. The winter monsoon usually lasts from November to March, characterized by strong northeasterly winds, colder sea surface temperatures (SSTs), wetter conditions and lower salinities (Fig. 1; see Supplementary Fig. S1). The summer monsoon, usually from May to September, is distinguished by reduced southwesterly winds, highest SST, lower precipitation and higher salinity (Fig. 1; see Supplementary Fig. S1). However, precipitation in northern Borneo is more evenly distributed across the annual cycle, less monsoonal and dominated by intraseasonal and interannual variability^16^. Intra-monsoon rainfall occurs in April and October in any given year, with the latter often leading to wetter conditions and lower salinities in the southern SCS^17^. The region’s climate variability is influenced by global interannual phenomena that include the El Niño-Southern Oscillation (ENSO), the Indian Ocean Dipole (IOD), the intraseasonal Madden-Julian Oscillation (MJO) and the Borneo Vortex^14,16^. In SEA, drier than normal conditions are associated with El Niño, whereas wetter than normal conditions tend to be associated with La Niña^14,18^. Apart from this general relationship, the ENSO-precipitation connectivity in SEA shows spatial and seasonal variation. The relationship between Malaysian precipitation and ENSO strengthens from September to March of the following year^19^. ENSO events can generate severe floods, droughts and drought-related forest fires, which can have devastating socio-economic and ecologic consequences^20–22^. It has been suggested that the Pacific Decadal Oscillation (PDO) induces decadal precipitation and salinity anomalies in the South China Sea (SCS) on top of interannual ENSO events^23^. Unfortunately, the instrumental data coverage for the Miri-Sibuti Coral Reefs National Park (MSCRNP), situated off the Sarawak coast, is poor. Therefore, the lack of seasonally resolved climatic baseline data limits our understanding of the spatio-temporal change of the coral reefs’ physical environment in MSCRNP, and its regional and large-scale drivers.

Massive scleractinian corals like *Porites* spp. are a key archive for past climate and environmental change, including SST and hydrologic balance, and can augment scarce instrumental data^24,25^. The Sr/Ca ratio in coral skeletons is considered to be the most reliable proxy for SST^26–30^, though some studies have shown it to be affected by biomineralization processes, so called “vital effects”, which can influence Sr/Ca ratios independently from water temperatures^31^. Another method is to reconstruct SSTs from coral δ^18^O^26^. The use of δ^18^O for paleothermometry was pioneered by Urey and Epstein, who demonstrated a reverse relationship between δ^18^O and temperature values^32–34^. Weber and Woodhead^35^ were the first to apply it to corals in 1972, and since then δ^18^O from coral skeletons has been widely used as a tracer for up to monthly resolved SST^36,37^. However, coral δ^18^O varies as a function of two major variables, temperature and isotopic composition of the seawater, with the latter being affected by rainfall, evaporation, advection of water masses and freshwater runoff^38–41^. As such, past changes in the hydrologic balance for inshore coastal waters can be provided by using paired Sr/Ca and δ^18^O analysis on the same samples. This pairing quantitatively separates the effects of SST from those of the ambient seawater’s oxygen isotopic composition on the skeletal δ^18^O and provides reconstructed δ^18^O_seawater (sw),_ related to changes in the hydrological balance and salinity^42–44^.

**Figure 2:**
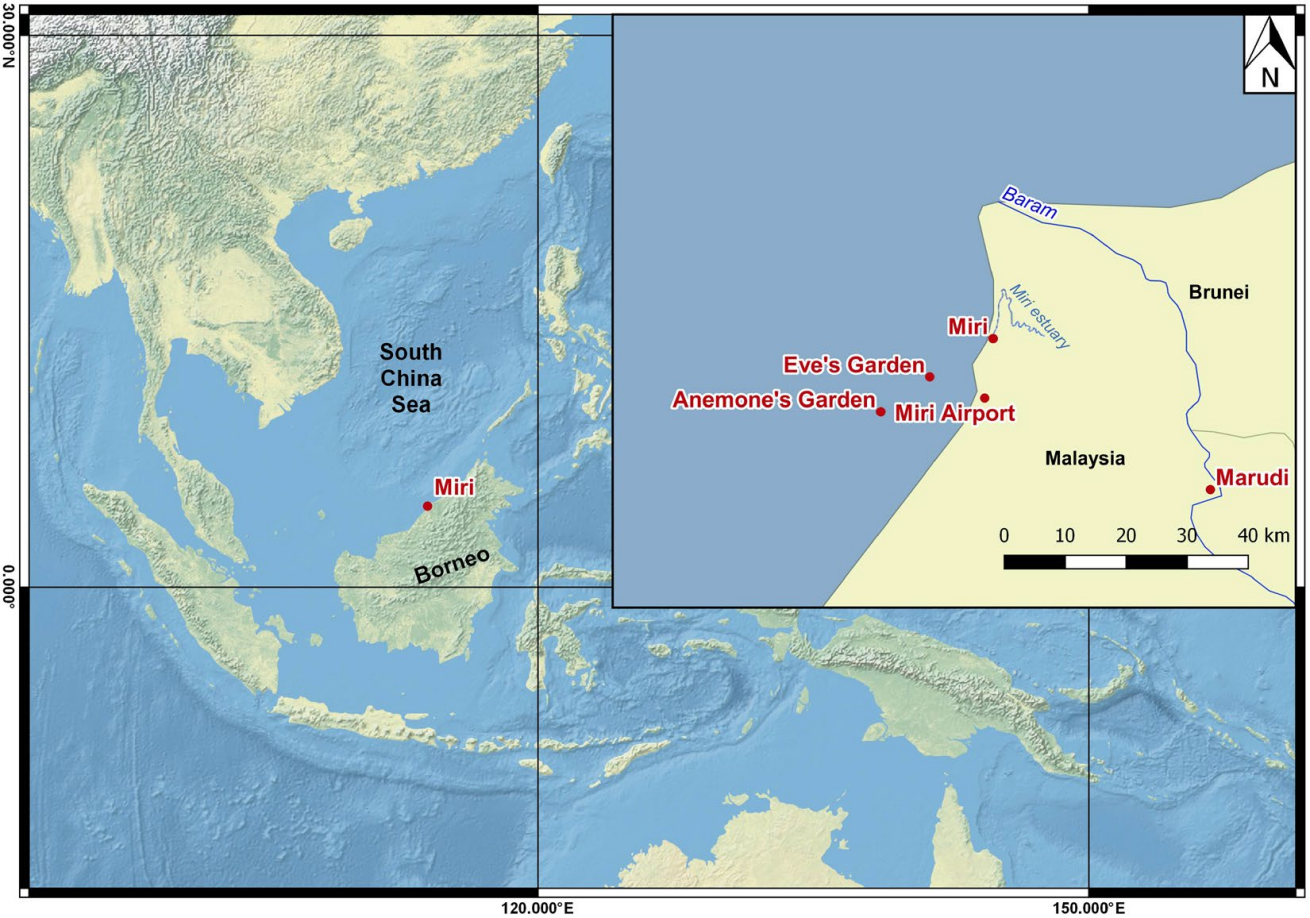
Map of South China Sea (SCS) with enlarged map of study area, showing locations of reefs Anemone’s Garden (AG) and Eve’s Garden (EG), Miri city, Miri airport weather station, Marudi river discharge station and the path of the Baram River. (Made with Natural Earth - Free vector and raster map data in QGIS 3.4 Madeira^89^).

The aim of this work was to develop a spatio-temporal reconstruction of SST, oxygen isotopic composition of seawater tracking sea surface salinity (SSS) and river runoff dynamics based on a multi-proxy analysis of coral cores from two sites in MSCRNP, Borneo (Malaysia). In September 2016 coral cores were retrieved from Eve’s Garden (EG) and Anemone’s Garden (AG). These two patch reefs lie along an inshore to offshore gradient and are therefore at different depths, distances from shore and distances from the Miri estuary and the Baram River mouths^45^ (Fig. 2; Table 1). Sr/Ca ratios in coral skeletons were utilized to reconstruct local SSTs. Combined Sr/Ca and oxygen isotope analysis enabled us to reconstruct the oxygen isotopic composition of the seawater related to changes in the hydrological balance and salinity, covering the past three decades. This allowed for an unprecedented comparison to instrumental data from both land and sea from 1982 to 2016. All coral proxies were validated against instrumental data products of SST, SSS, river discharge and precipitation. Our results, were further investigated for signals of large-scale climatic teleconnections in northern Borneo, related to ENSO and the PDO.

**Table 1:** Reef locations in relation to coastline and rivers, coral colony depths, colony heights and coordinates.

	Shore distance [km]	Baram distance [km]	Miri River distance [km]	Colony top depth [m]	Colony height (m)	Coordinates [latitude, longitude]
Eve’s Garden (EG)	7.3	28.8	10.8	5	1.6	4°20′36.05″N 113°53'53.94″E
Anemone’s Garden (AG)	11.7	36.3	20	8.5	1.5	4°17′31.81″N 113°49′33.28″E

## Results

### Monthly interpolated geochemical proxy time series

**Figure 3:**
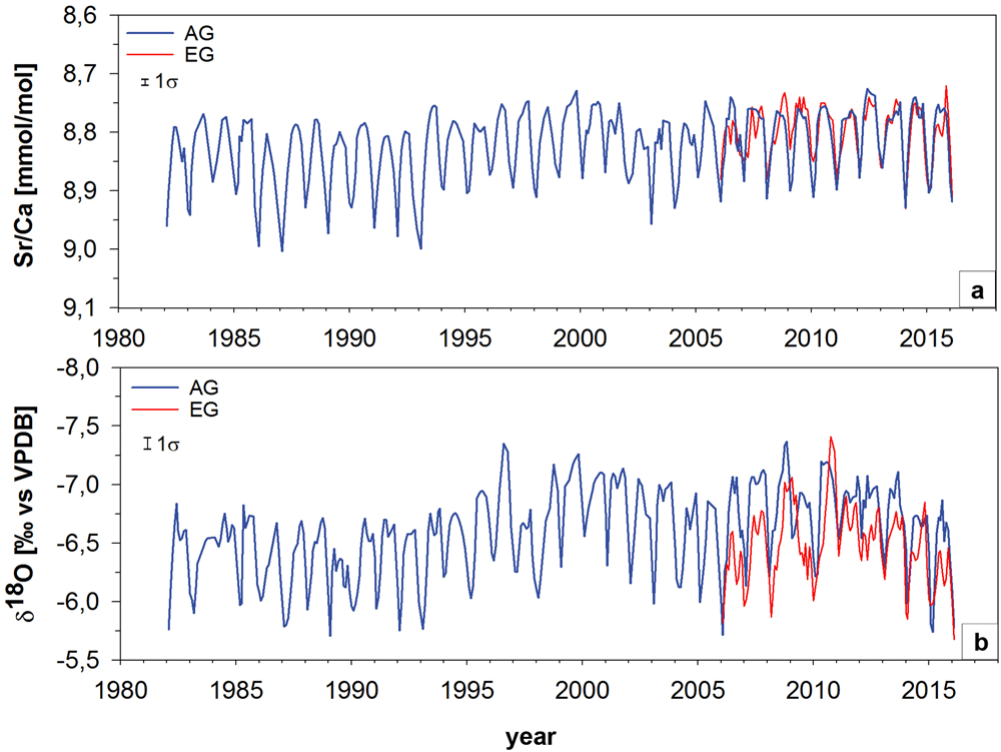
Monthly interpolated time series of Sr/Ca and δ^18^O data from AG (blue) and EG (red) cores. Y-axes are reversed from high to low values. Error bars represent 1σ.

The monthly interpolated time series of AG and EG coral Sr/Ca and δ^18^O revealed a distinct seasonality with higher values during the winter season (December to February) and lower values in the summer season (June to August) (Fig. 3). Spatial comparisons of the two reefs between 2006 and 2016 found no difference between Sr/Ca ratios at AG and EG (see Supplementary Table S1). For mean coral δ^18^O, AG had lower values than EG (Fig. 3; see Supplementary Table S1). Linear correlations between Sr/Ca and δ^18^O in both AG and EG were highly significant (p < 0.0001). The correlation between Sr/Ca and δ^18^O at AG (r = 0.81) was stronger than at EG (r = 0.62).

AG Sr/Ca ratios displayed an overall decrease of 0.06 mmol/mol from 1982 to 2016. Seasonal Sr/Ca amplitudes also decreased at AG from approximately 1993, although there were higher winter values in some years (e.g. 2014 to 2016; Fig. 3a). There was no trend in Sr/Ca at EG between 2006 and 2016. The AG δ^18^O record showed an overall decrease of 0.45‰ and had stronger interannual and decadal variability than the Sr/Ca record (Fig. 3b). In the long term, the annual mean δ^18^O values exhibited slight shifts on an approximately decadal time scale with a prominent shift to lower values in the mid to late 1990s (Fig. 3b). From 1982 until 2006, δ^18^O at AG decreased by 0.46‰. Yet from 2006 to 2016, the mean δ^18^O at AG increased (AG = 0.2‰, p = 0.04) while EG showed no significant trend (EG = 0.14‰, p = 0.12).

### Monthly calibration of Sr/Ca with sea surface temperature (SST) and SST variability

Ordinary least squares regressions between coral Sr/Ca and AVHRR-OISSTv2^46,47^ were used to reconstruct absolute SST from coral Sr/Ca (Table 2). Our observed strong correlation between Sr/Ca and SST at the study sites indicates that growth rate and vital effects had an insignificant effect on skeletal Sr/Ca. The local proxy-SST relationships varied between −0.043 mmol/mol °C^−1^ in AG and −0.039 mmol/mol °C^−1^ in EG, which is in agreement with the range of published slopes^26,29^ that lie between −0.04 and −0.08 mmol/mol °C^−1^. Our regression slopes were also comparable to other coral core studies in the northern SCS^48–50^. The root mean square error (RMSE) of individual monthly interpolated reconstructed SST for the AG and EG record was 0.64 °C and 0.55 °C, respectively.

**Table 2:** Ordinary Least square linear regressions of Sr/Ca against NOAA OISSTv2^46,47^ for each coral core, yielding calibration equation for coral-derived SST reconstructions.

	m	SE m	b	SE b	R²	p	error (°C)	SE of estimate	n
**Sr/Ca vs NOAA OISSTv2 Proxy** = m (slope) *SST+b (y-intercept)									
**AG**	−0.043	0.002	10.068	0.049	0.611	<0.0001	0.64	0.035	409
**EG**	−0.039	0.003	9.923	0.079	0.633	<0.0001	0.55	0.027	121

**Figure 4:**
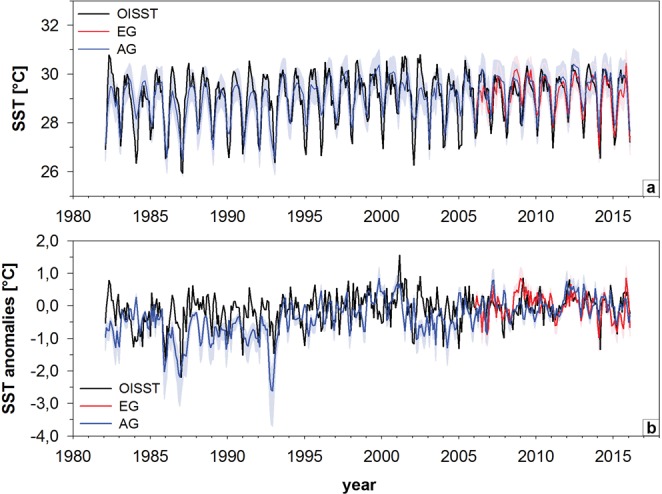
(**a**) Monthly interpolated time series of reconstructed AG (blue) and EG (red) Sr/Ca-SST and instrumental 0.25° gridded NOAA AVHRR-OISSTv2^46,47^ (black). Shading indicates reconstruction uncertainty of Sr/Ca-SST (root mean square error; RMSE). (**b**) Monthly interpolated AG (blue) and EG (red) Sr/Ca-SST anomalies and NOAA AVHRR-OISSTv2 (black) anomalies relative to 2006-2015 means. Shading indicates uncertainties in AG (blue) and EG (red) Sr/Ca-SST anomalies using varying regression slopes (−0.043 mmol/mol °C^−1^ local slope; −0.06 mmol/mol °C^−1^ and −0.084 mmol/mol °C^−1^ ^26,29^.

Reconstructed monthly interpolated SST across the two sites varied between 26.5 °C and 30.5 °C in Sr/Ca-SST and between 25.9 and 30.8 °C in AVHRR-OISSTv2 data for the 0.25° × 0.25° grid^46,47^. Both Sr/Ca-SST (0.85 ± 0.13 °C) and instrumental SST (0.41 ± 0.18 °C) showed a long-term warming trend (Fig. 4a). AVHRR-OISSTv2 had larger seasonal amplitudes, especially before 2005, with lower winter SSTs and higher summer SSTs (Fig. 4a). Within the reconstructed SST data, seasonal amplitudes declined after 1993, with a shift to higher winter temperatures and smaller seasonal amplitudes (see Supplementary Fig. S2). Occasionally, winter Sr/Ca-SSTs reached values similar to pre-1993, for instance between 2002–2003 and 2014–2016 (Fig. 4b). The coral-derived SST anomalies in both AG and EG relative to the 2006 to 2016 mean (years of coral core overlap) mirrored the interannual variations of instrumental SST anomalies well (Fig. 4b). Both AVHRR-OISSTv2 and our coral Sr/Ca-SST records showed no significant correlation with the Niño3.4 index, yet both the AVHRR-OISSTv2 and AG Sr/Ca-SST had significant relationships with Niño4 in Boreal winter to spring (see Supplementary Table S3).

AG Sr/Ca anomalies relative to a 1982 to 2016 mean were converted to SST using the local calibration slope (−0.04 mmol/mol °C^−1^; Fig. 4b), a mean slope of −0.06 mmol/mol °C^−1^ and a slope of −0.08 mmol/mol °C^−1^ to estimate an error range of the calibration^26,29^. The coral-derived SST anomalies followed the trends of instrumental SST anomalies reasonably well (Fig. 4b). However, the regression slopes of −0.08 mmol/mol °C^−1^ and −0.06 mmol/mol °C^−1^ resulted in SST anomalies that closely aligned with AVHRR-OISSTv2, while the locally derived −0.04 mmol/mol°C^−1^ slope estimate deviated the most. Between 1982 and 1994, the AG Sr/Ca-SST anomalies (relative to the 1982–2016 mean) were 0.5 °C lower than between 1994 and 2016. Sr/Ca-SST anomalies fluctuated around the 1982–2016 mean from 2002–2005, as well as from 2009–2010.

### δ^18^O_sw_ reconstruction and relationship with salinity, rainfall and river discharge

**Figure 5:**
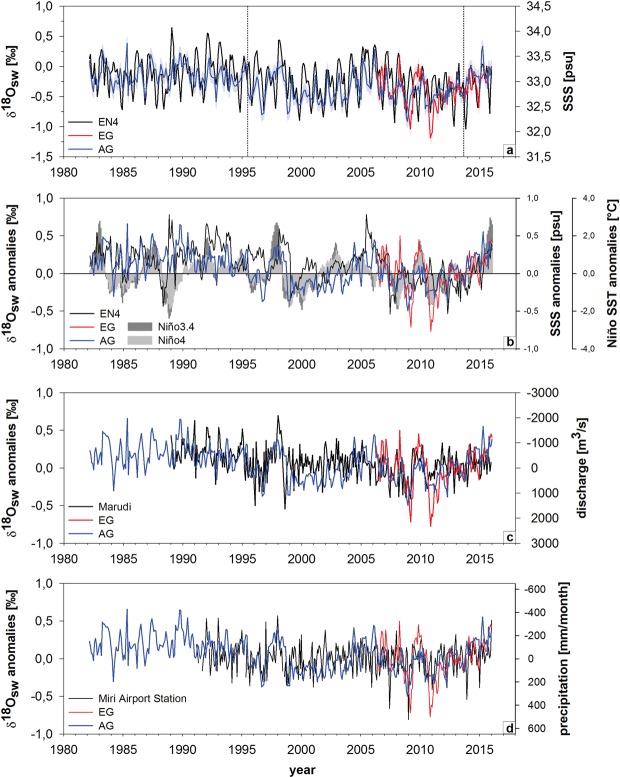
(**a**) Monthly interpolated time series of reconstructed δ^18^O_sw_ (red = EG, blue = AG) and EN4 SSS^66^. δ^18^O_sw_ reconstruction is based on coral δ^18^O in ‰ vs. VPDB. Uncertainties in reconstructed δ^18^O_sw_ (1σ) indicated by shading. Change-points in mean and variance of reconstructed δ^18^O_sw_ indicated by stippled lines. (**b**) Coral-derived δ^18^O_sw_ anomalies (in ‰ vs. VPDB) compared to EN4 SSS anomalies^66^ and Niño3.4/Niño4 SST anomalies^51^ relative to 2006–2015 means. (**c**) Coral-derived δ^18^O_sw_ anomalies compared to Marudi station discharge^15^. (**d**) Coral-derived δ^18^O_sw_ anomalies compared to precipitation anomalies at Miri airport weather station^84^.

Mean reconstructed δ^18^O_sw_ between 2006 and 2016 was similar at EG (−0.38 ± 0.26‰) and AG (−0.36 ± 0.22‰) (Fig. 5a; see Supplementary Table S1). Mean annual and detrended δ^18^O_sw_ in AG and EG were highly correlated (r = 0.93, p < 0.001). There was also a positive and significant correlation between mean annual reconstructed δ^18^O_sw_ and EN4 SSS at AG (r = 0.72, p < 0.001) and EG (r = 0.68, p = 0.046). The correlation remained significant for seasonal mean values (see Supplementary Table S4). δ^18^O_sw_ in AG and EG mostly mirrored the long-term and interannual to decadal variability of the EN4 SSS data between 1982 and 2016, with higher values matching higher salinity (Fig. 5a). EG δ^18^O_sw_ values showed a higher variability in individual years than AG between 2006 and 2016. AG δ^18^O_sw_ between 1982 and 2016 indicated a decrease of 0.27 ± 0.04‰ (p < 0.001), while EN4 SSS decreased by 0.32 ± 0.06 psu (p < 0.001). Both AG and EG δ^18^O_sw_ displayed decadal variability between 2006 and 2016 in agreement with EN4 SSS (Fig. 5a). Mean annual δ^18^O_sw_ at AG and EG, and EN4 SSS were negatively correlated with the local precipitation and Marudi river discharge. All correlations were statistically significant with the exception of EN4 SSS with Marudi discharge (Table 3).

**Table 3:** Correlation coefficients and p-values between mean annual and detrended coral-derived δ^18^O_sw_ with EN4 salinity^66^, Miri station precipitation^84^ and Marudi station river discharge^15^.

Record	Time	r	p
**EN4 SSS vs. δ**^**18**^**O**_**sw**_			
AG	1989–2016	0.72	<0.001
EG	2006–2016	0.68	0.046
**Miri station precipitation vs. δ**^**18**^**O**_**sw**_ **and SSS**			
AG	1991–2016	−0.68	<0.001
EG	2006–2016	−0.86	<0.003
EN4 SSS	1991–2016	−0.53	0.009
**Marudi river discharge vs. δ**^**18**^**O**_**sw**_**, EN4 SSS and precipitation**			
AG	1989–2016	−0.61	0.002
EG	2006–2016	−0.71	0.033
EN4 SSS	1989–2016	−0.29	0.166
Precipitation	1991–2016	0.92	<0.001

Anomalies of reconstructed δ^18^O_sw_ and EN4 SSS were calculated relative to the 2006 to 2016 means (Fig. 5b). With some exceptions, AG’s δ^18^O_sw_ followed the interannual variability and trends observed in EN4 SSS since 1982 (Fig. 5b). The comparison of the anomalies with ENSO indices Niño3.4 and Niño4^51^ showed good agreement between the coral-derived δ^18^O_sw_ (AG and EG) and ENSO indices. For example, high δ^18^O_sw_ matched the 1997/98 El Niño and was followed by a 1998/99 La Niña phase and a shift to low δ^18^O_sw_ in the coral record (Fig. 5b; see Supplementary Fig. S2). Generally, both AG and EG recorded similar interannual variability with slightly larger magnitudes for EG (Fig. 5b). Both AG and EG δ^18^O_sw_ also largely mirrored interannual variations in river discharge and precipitation anomalies (Fig. 5c,d). Marudi discharge indicated a significant increase of 641 ± 121 m^3^/s between 1989 and 2015 (p < 0.001) while Miri precipitation recorded a non-significant increase of 56 ± 38 mm month^−1^ (p = 0.15) between 1992 and 2016.

### Regional and large-scale teleconnection of reconstructed δ^18^O_sw_ with climate parameters

Coral δ^18^O_sw_ at AG and EG, precipitation from the local weather station, Marudi river discharge and EN4 SSS were correlated with ENSO SST indices Niño3.4 and Niño4^51^ and the PDO^52^ (Table 4). All correlations with ENSO indices, exhibited statistical significance averaged over a 3-month period (Table 4). All data, except for precipitation and river discharge (negative correlation), were significantly positively correlated with Niño3.4 and Niño4 (Table 4). Miri precipitation and AG δ^18^O_sw_ had the highest correlations in the February to April (FMA) season, while EN4 SSS had it between March to May (MAM) (Table 4). EG δ^18^O_sw_ showed the highest correlations with Niño3.4 and Niño4 between November to January and December to February, respectively (Table 4). Miri precipitation and Marudi river discharge indicated the highest, statistically significant (p < 0.05) correlations between February and April and August to October (Table 4).

**Table 4:** Correlation coefficients of Miri rainfall^84^, Marudi river discharge^15^, gridded salinity (EN4 SSS^66^) and δ^18^O_sw_ of AG and EG with Niño3.4^51^, Niño4^51^ and PDO^52^ indices averaged over 3 months. All data detrended with 95% confidence intervals indicated in last column. P-values (p) and numbers of years (n) for correlations indicated. Correlations made with KNMI Climate Explorer^80^.

		Months	r	p	n	95% CI
**Averaged over 3 months**						
Miri precipitation	NINO3.4	Feb–Apr	−0.78	0.001	123	−0.93; −0.61
	NINO4	Feb–Apr	−0.74	0.001	23	−0.87; −0.56
	PDO	Feb–Apr	−0.68	0.001	24	−0.82; −0.51
Marudi river discharge	NINO3.4	Feb–Apr	−0.77	0.001	27	−0.89; −0.54
	NINO4	Feb–Apr	−0.6	0.001	27	−0.77; −0.42
	PDO	Feb–Apr	−0.61	0.001	27	−0.82; −0.26
EN4 SSS	NINO3.4	Mar–May	0.52	0.001	35	0.18; 0.69
	NINO4	Mar–May	0.41	0.016	35	0.08; 0.60
	PDO	Apr–Jun	0.41	0.016	35	0.15; 0.66
AG δ^18^O_sw_	NINO3.4	Feb–Apr	0.56	0.001	34	0.31; 0.76
	NINO4	Feb–Apr	0.63	0.001	34	0.35; 0.80
	PDO	Apr–Jun	0.57	0.001	34	0.31; 0.73
EG δ^18^O_sw_	NINO3.4	Nov–Jan	0.78	0.007	10	0.29; 0.93
	NINO4	Dec–Feb	0.79	0.007	10	0.25; 0.93
	PDO	Feb–Apr	0.79	0.012	10	0.61; 0.96

Seasonal δ^18^O_sw_ at AG (April to June) and EG (February to April) were positively correlated with the PDO^52^ between 1982 and 2016 and 2006 to 2016, respectively (Table 4). The same holds for mean annual δ^18^O_sw_ at AG (r = 0.56, p = 0.002) and EG (r = 0.75, p = 0.02). Marudi discharge (1989–2015) and Miri station precipitation (1992–2016) were significantly negatively correlated with the PDO on seasonal time scales (February to April; Table 4). The correlation was positive and lower between EN4 SSS and the PDO, yet significant (p < 0.05) for both seasonal (Table 4) and mean annual time scales (r = 0.38, p = 0.038).

**Figure 6:**
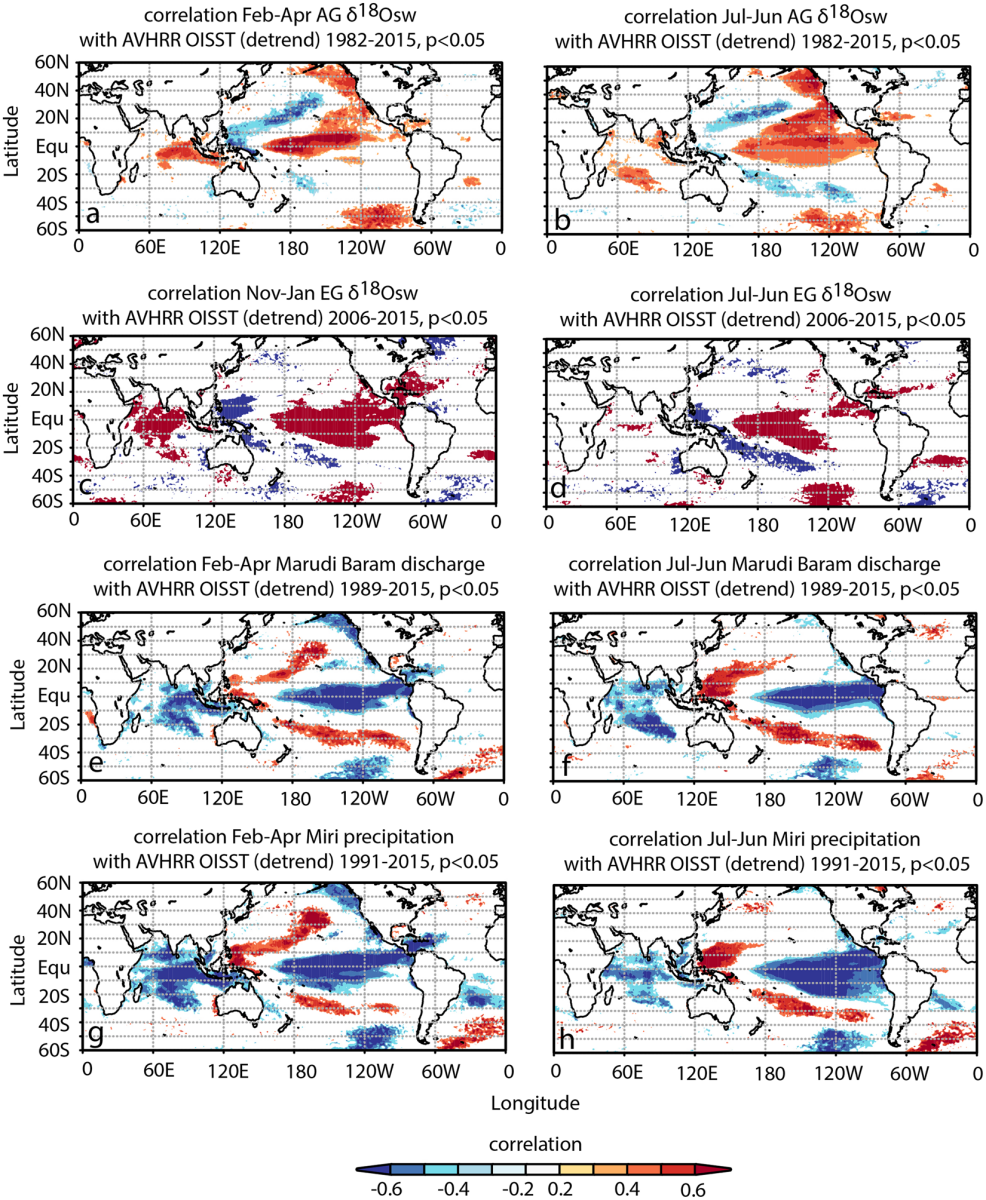
Spatial correlation of AG δ^18^O_sw_ with NOAA OISST 0.25° ^46,47^, averaged over 3 months (**a**) and 12 months (**b**), both for 1982–2015. (**c**,**d**) same as (**a**,**b**), yet for EG δ^18^O_sw_, (**e**,**f**) Spatial correlation of local precipitation^84^ with NOAA AVHRR-OISSTv2 0.25°^46,47^, averaged over 3 months and 12 month. (**g**,**h**) Spatial correlation of Marudi river discharge with NOAA AVHRR-OISSTv2 0.25°^46,47^, averaged over 3 months and 12 months. Spatial correlations only shown for season and year of highest correlation with ENSO indices. Only correlations with p < 0.05 are colored. Computed with KNMI Climate Explorer^80^.

Spatial correlations of AG and EG δ^18^O_sw_ and local precipitation against OISSTv2 displayed the typical ENSO “horseshoe” SST pattern at both seasonal and mean annual time scales (Fig. 6a–d). AG and EG δ^18^O_sw_ were positively correlated with the central and eastern Pacific SST and negatively correlated with the northwestern, central and southwestern Pacific SST. Further there was a positive correlation with the northeastern and central Indian Ocean SST and negative correlation with the southeastern Indian Ocean SST (Fig. 6a–d).

Correlations of local rainfall (Fig. 6e,f) and river discharge (Fig. 6g,h) indicated the same pattern in the Pacific, except with an inverse relationship between precipitation, river discharge and SST. There was significant negative correlation between Miri precipitation and river discharge with northern and central Indian Ocean SST for both seasonal (FMA) and annual means (Fig. 6e,f). Additionally, seasonal means of local precipitation and river discharge displayed positive correlation with southeast Indian Ocean SST. The spatial correlations of Miri station precipitation with SST also revealed the “horseshoe” ENSO SST pattern (Fig. 6g,h).

## Discussion

Our observed strong correlation between Sr/Ca and SST at the study sites indicates that growth rate and vital effects had an insignificant effect on skeletal Sr/Ca. The Sr/Ca-SST reconstructions from the AG coral core record suggested an increase of 0.26 ± 0.04 °C per decade since 1982, with more pronounced warming during the winter (0.27 ± 0.05 °C per decade) than the summer months (0.19 ± 0.03 °C per decade) (see Supplementary Fig. S2). The gridded SST data indicated a range in warming trends between 0.12 ± 0.04 (0.25 × 0.25 °AVHRR-OISSTv2^46,47^) and 0.17 ± 0.05 °C per decade (1 × 1° OISSTv2^46,47^). However, the relatively high warming trends in coral-derived SST conformed with other studies in the region. For example, SST reconstructions from a *Porites* in the Nansha Islands in the southern SCS from 1951 to 1998 indicated an increase of 0.21 °C per decade^53^. The work of Heron *et al*.^54^ specifically investigated SST trends from 1985 to 2012 in several reef regions of the world. They stated that warming of reef waters was distinctly higher than reported for ocean waters and increased by 0.21 °C per decade in the reef waters surrounding Sulawesi, Indonesia. This study has a comparable time frame for the same region with this study thereby validating the magnitude of the trend in AG Sr/Ca-SST. The more pronounced warming during the winter months was also a common observation of several studies from the SCS. For example Bao and Ren^55^ calculated an increase of 0.23 °C per decade (1962 to 2011) for winter SSTs and 0.16 °C per decade for summer SSTs. The stronger increase in winter SST might be related to a weakening of the East Asian Winter Monsoon (EAWM)^48,55,56^, which results in less transport of cooler extratropical waters from the north through the Taiwan Strait fed by the Western Pacific. Thus, the West Pacific seems to be the dominant factor controlling SST variations in the SCS, especially in the southern section, where the monsoonal influence is generally lower than in the north^48,57^. On interannual time scales, ENSO dominates the SST response in the SCS with warm anomalies in the mature phase of El Niño (December to February) extending into spring (March to May)^58,59^. However, correlations of AVHRR-OISSTv2 near our study sites and our Sr/Ca records with the Nino3.4 index were not significant during the Boreal winter, while those with the Nino.4 index were weak, yet significant between January and April. This indicates that the southern SCS is indeed most sensitive to western Pacific SST and circulation anomalies as proposed by Juneng and Tangang^58^. Decadal changes superimposed on the overall rising temperature trend is a common observation in coral records from the SCS^48,60^. Drivers of these decadal shifts could include the PDO or Interdecadal Pacific Oscillation (IPO^61^). AVHRR-OISSTv2 did indicate weak, yet significant correlations with the PDO on seasonal timescales (r = 0.41, p = 0.017), yet not on mean annual time scales. Correlations between the PDO and AG and EG Sr/Ca were not significant. Thus, the influence of the PDO on SST in the southern SCS remains uncertain.

Coral-derived δ^18^O_sw_ from AG and EG exhibited significant correlation with ocean SSS (offshore grid), local precipitation and river discharge suggesting that the coral record can provide reliable information on changes in the hydrological balance in reef waters. Regional monitoring of oxygen isotopes in rainfall between 2004 and 2011 upstream and downstream in the Baram catchment (Mulu and Lambir Hills National Park) indicated a clear inverse relationship with the amount of rainfall^62,63^. Therefore, δ^18^O of seawater around Miri is influenced by the balance between precipitation and evaporation (the P-E balance), and freshwater runoff. Thus, sea surface salinity, which is also largely modified by P-E balance and riverine freshwater runoff, is linearly related to δ^18^O_sw_^39,64,65^. In the southern part of the SCS precipitation exceeds evaporation^57^. EN4 SSS^66^ for the grid closest to our coral reefs showed significant correlations with precipitation at Miri (r = −0.53, p < 0.009) yet not with river discharge at Marudi (r = −0.29, p = 0.166). The modest correlations of EN4 SSS^66^ with precipitation and river discharge could be explained by salinity being averaged over a larger offshore oceanic grid (3.5–4.5°N, 112.5–113.5°E) that is some distance from the rainfall station and rivers. However, correlations of coral δ^18^O_sw_ from AG and EG with the local precipitation (AG: r = −0.68, EG: r = −0.83, p < 0.003) indicated a significant inverse relationship between the reefs’ seawater δ^18^O and the seasonal cycle of local rainfall, with lowest δ^18^O_sw_ during the winter monsoon. The same holds for correlations with river discharge at both EG and AG. Mean δ^18^O_sw_ in EG and AG did not differ, despite EG’s closer proximity to the river mouths. Exceptionally lower peaks in EG than AG, for example in the winter months of 2008/9 and 2010/11, could be attributed to heavy rainfall and high runoff events, which are typically more pronounced closer to the river source. The AG δ^18^O_sw_ record for 1982 to 2016 was also in good agreement with trends and interannual to decadal variability in oceanic salinity data with few exceptions. The same holds for correlations between AG δ^18^O_sw_ and river discharge between 1989 and 2016. As such, both AG and EG reliably recorded the regional P-E balance and river discharge. Yet, isotope analysis and salinity measurements of water samples on a transect from the river mouth to the reefs would provide a better understanding of riverine impact in future studies.

Both the gridded SSS data and the coral-derived δ^18^O_sw_ displayed an interdecadal shift in the mid-1990s superimposed on the interannual ENSO variability (see Supplementary Fig. S3). Several studies in the SCS attributed these interdecadal shifts to the PDO. Zeng *et al*.^67^ observed a salinification trend in the SCS from approximately 1973 to 1993, a freshening from 1993 to 2012 and again salinification from 2012 to the present. These trends were also observed in the coral-derived δ^18^O_sw_ (salinification 1982 to 1994, freshening 1995 to 2013, salinification 2014 to 2016) and gridded SSS data in MSCRNP (Fig. 5; see Supplementary Fig. S3). Both AG and EG δ^18^O_sw,_ as well as precipitation and river discharge, indicated robust relationships with the PDO, while EN4 SSS showed weaker, yet statistically significant relationships (Table 4). It has been suggested that the positive PDO phase induces dry sinking air along with reduced rainfall and vice versa in the negative phase in the SCS^23^. In addition, salinification during positive PDO is further enhanced due to a directional switch in the horizontal current through the Luzon Strait, leading to the advection of more saline waters into the SCS^67^. In line with these findings, Deng *et al*.^68^ showed significant correlation between δ^13^C and δ^18^O from a coral proxy record in the northern SCS and the PDO. They suggest that PDO is causing precipitation anomalies on interdecadal scales, influencing the hydrological balance in the region due to changing rainfall and river runoff dynamics. It is therefore likely that Pacific decadal variability has influenced long-term salinity trends in the SCS. Therefore, our coral δ^18^O_sw_ records should be extended in future studies to cover the past two centuries to assess the stability of the ENSO teleconnection and decadal shifts related to the PDO identified in our study.

The correlations of precipitation and the coral δ^18^O_sw_ in Miri with ENSO indices demonstrated a robust relationship between the ENSO phenomenon and hydroclimate in northern Borneo. The spatial correlations resembled the ENSO “horseshoe” SST pattern in agreement with previous studies^19,58^ (Fig. 6). The El Niño events in 1983, 1988, 1992, 2010 and 2014–2016 all showed consistent positive δ^18^O_sw_ and salinity anomalies indicating reduced runoff or P-E testifying to the paramount impact of ENSO on SEA drought. The Marudi river discharge and Miri precipitation records confirmed the reduction in freshwater runoff from the Baram River since 1989 and precipitation since 1992 during all El Niño years. The comparison of coral-derived δ^18^O_sw_ anomalies with the SST anomalies in the Niño 3.4 and Niño 4 regions also displayed good agreement, with a particularly striking shift in δ^18^O_sw_ anomalies matching the 1997/98 El Niño followed by 1998/99 La Niña. The latter is not surprising since winter precipitation in Borneo was reduced by more than 50% during the 1997/98 El Niño event^69^. In addition, 2005 appeared to show an El Niño-like response in our coral δ^18^O_sw_ records, SSS, precipitation and river discharge. 2005 was classified as a weak El Niño year in the Niño 3.4 and Niño 4, yet as El Niño Modoki^59^ or central Pacific El Niño, respectively. The response in 2005 could be an indicator of different local signals of Borneo’s hydroclimate to varying spatial expressions of ENSO SST anomalies as suggested by Tan *et al*.^59^. In contrast, during La Niñas of 1996, 1999–2001, 2008 and 2011 negative δ^18^O_sw_ were observed in agreement with lower SSS and higher river discharge. Several other paleoclimate studies in the SCS on corals from the northern SCS^48^, a speleothem record from northwestern Borneo^70^ and the δ^18^O of rainfall interannual anomalies at Gunung Mulu and Lambir Hills National Parks in northern Borneo have also shown clear ENSO impacts^62,63^. The proxy records together with the rainfall isotope data support the observed pattern of anomalously low precipitation and droughts in the Maritime Continent during El Niño^71,72^. This drying across the Maritime Continent is associated with warm SST anomalies during the El Niño mature phase causing an eastward shift in convection^72^. During La Niña convection strengthens in the Maritime Continent, causing anomalously high precipitation and floods. In northern Borneo the impact of ENSO starts in Boreal autumn and persists through Boreal spring with strongest responses in the winter season^19,58,73,74^, which is in accordance with the results from the coral records. The peak drying response to El Niño in northern Borneo in winter and spring is associated with the northeastward shift in intraseasonal convective activity into the northern hemisphere and the development of an anticyclone in the West North Pacific^19,58^. Tangang and Juneng^19^ suggested that the correlation between northern Borneo precipitation and ENSO in December to February is not a direct response to the eastern Pacific pole of canonical ENSO, but rather related to a dipole in SST strengthening the western Pacific arm of the ‘horseshoe’ SST pattern generated by ENSO. This interpretation is in agreement with our results showing highest correlations of coral δ^18^O_sw_ (also precipitation, Marudi discharge and Miri precipitation) in winter/early spring (February to April) with northwestern Pacific SST east of the Philippines (Fig. 6). Thus, coral δ^18^O_sw_ proves to be a reliable tracer of ENSO impacts on precipitation and river discharge in the region. In addition, our results provided clear evidence for ENSO-driven synchrony of climate impacts on both terrestrial and marine environments in northern Borneo. Furthermore, our findings provided invaluable data to better understand ENSO and PDO impacts on the southern SCS, but also for reef monitoring and protection, since both thermal stress and freshwater flux and related sediment transport may put coral reefs increasingly at risk along the northern Borneo coast in the coming decades. Future studies will expand the proxy reconstructions for this region both temporally and spatially to test for long-term stability of ENSO and PDO relationships with the Asian-Australian monsoon and its potential modification by recent global warming.

## Methods

### Coral sampling and core treatment

In September 2016, coral cores were retrieved from two reef sites in MSCRNP, at Sarawak’s northern coast (Fig. 1). Fieldwork was approved by the Sarawak Forestry Commission (permit no (61)/JHS/NCCD/600-7/2/107) and methods were executed in accordance with the approved guidelines and regulations. Coral cores were imported under CITES licence number 002259. The two reefs, Eve’s Garden (EG) and Anemone’s Garden (AG), lie along an inshore to offshore gradient and at different depth, distance from shore and distance from river mouths (Fig. 1; Table 1)^45^. Coral cores were drilled using a SCUBA tank driven pneumatic drill (Silverline Air Drill Reversible), with a diamond-coated drilling head. Core segments of approximately 30 cm in length and 4 cm in diameter were obtained along the central growth axis of massive, dome-shaped *Porites* sp. colonies. Cores were then sectioned into 7–8 mm thick slabs longitudinally, along the axis of growth and cleaned following the chemical treatment method developed by Nagtegaal *et al*.^75^. Slabs were submerged for 24 hours in a bath of sodium hypochlorite solution NaOCl (with 6–14% active chlorine) diluted to a 1:1 ratio with distilled water. Thereafter, slabs were rinsed three times in an ultrasonic bath filled with distilled water for 10 min, with water exchanged after every run. In between turns coral segments were blown with compressed air to remove stray particles. Finally, slabs were dried in a drying cabinet at 50 °C for 24 to 48 h. X-ray radiography was used to visualize annual density bands and to determine the sampling path along the major growth axis (see Supplementary Fig. S4). Samples for geochemical analysis were drilled every 1.1 mm, using a diamond coated dental drill. The number of samples was n = 271 for Anemone’s Garden and n = 103 for Eve’s Garden. The sample powder was then split for stable isotope and Sr/Ca measurements.

### Sr/Ca measurements and SST reconstruction

Sr/Ca ratios were measured at the University of Kiel with a simultaneous inductively coupled plasma optical emission spectrometer (ICP-OES, Spectro Ciros CCD SOP), following a combination of the techniques described by Schrag^76^ and de Velliers^77^. Sr and Ca were measured at their 421 and 317 nm emission lines, respectively. 175 ± 25 µg of coral powder was dissolved in 1 ml nitric acid (HNO_3_ 2%). Prior to analysis, this solution was further diluted with 4 ml HNO_3_ 2% to a final concentration of approximately 8 ppm. An analogously prepared in house standard (Mayotte coral) was measured after each sample batch of 6 samples to correct for drift effects. The international reference material JCp-1 (coral powder) was analysed at the beginning and end of every measurement run. Internal analytical precision based on replicate Sr/Ca measurements was 0.008 mmol/mol (1σ) or 0.08%. Average Sr/Ca value of the JCp-1 standard from multiple measurements on the same day and on consecutive days was 8.831 mmol/mol with 0.085% relative standard deviation (RSD). The comparison to the certified Sr/Ca value of 8.838 mmol/mol^78^ with an expanded uncertainty of 0.089 mmol/mol indicates a high external precision of <0.08%.

SST reconstructions in this study are based on calibrations of the coral Sr/Ca ratios with the OISSTv2 data. The relationship between the skeletal Sr/Ca and the instrumental data was quantified by (OLS) regression^79^.

1 $$Sr/C{a}_{coral}=m\times SS{T}_{recon.}+b$$

The error estimate for absolute SST reconstructions was calculated with the mean squares of the residuals of the inverted Eq. 1 ^41^.

The calculation of Sr/Ca-SST anomalies is based on relative changes in SST in comparison to a climatological mean. This method was used to eliminate errors associated with absolute SST reconstructions, since SST anomalies are independent from the intercept of the calibration. Sr/Ca anomalies for the AG record were calculated relative to the 1983 to 2015 mean, and converted to SST based on a range of published slopes to indicate uncertainties of the estimate^26,29^. Anomalies for the shorter records starting in 2006 of AG and EG were calculated relative to the average of 2006 to 2015. Calculation of anomalies was carried out with the KNMI Climate Explorer^80^. Based on empirical studies by Corrège^26^ and Gagan *et al*.^29^, the slopes of −0.06 mmol/mol °C^−1^ and −0.084 mmol/mol °C^−1^ were used for the conversion of Sr/Ca anomalies to SST next to local calibration slopes.

### Oxygen isotope measurements

Stable oxygen and carbon isotope measurments were performed at the Museum für Naturkunde, Leibniz-Institut für Evolutions- und Biodiversitätsforschung, Berlin (Germany). 200 ± 50 µg of coral powder from each sample was put into clean 10 ml exetainers. After being sealed with a septum cap, the remaining air was flushed out of the vessel with helium gas for 6 min at a flow of 100 ml/min. This process was followed by an injection of 30 µl of anhydrous phosphoric acid (H_3_PO_4_) through the septum. Each sample had a reaction time of approximately 90 min at 50 °C before measurement. Oxygen and carbon isotopic composition of the reaction product (CO_2_) was measured using a Thermo Finnigan GASBENCH II coupled with a Thermo Finnigan DELTA V isotope-ratio mass spectrometer (IRMS). Pure CO_2_ was used as reference gas for the measurements. The reference gas was calibrated against the VPDB (Vienna Pee Dee Belemnite) standard by using the IAEA (International Atomic Energy Agency) standards NBS18 and NBS19. Isotope values are shown in the conventional delta notation (δ^18^O) in per mil (‰) versus VPDB. The internal analytical precision based on replicate measurements of the reference gas was <0.05‰. The reproducibility of replicated lab standards (pulverized limestone) was generally <0.1‰ (1σ) for both carbon and oxygen isotopes.

### Age model

Age-depth models for each core were established using the software AnalySeries2.0^81^ and the NOAA AVHRR-OISSTv2 High Resolution Dataset for a 0.25° grid^46,47^. The chronology was generated based on the pronounced seasonal cycle of Sr/Ca ratios. The highest Sr/Ca values of each cycle were assigned to the coldest month from the instrumental data for each year. In cases of clear low peaks in the Sr/Ca ratios, these were assigned to the warmest months. The starting year was assigned to 2016, since the cores were drilled from living colonies. First, age assessments for all samples between the anchor points were obtained by linear interpolation in AnalySeries2.0^81^. In a second step, the sample data were interpolated to 12 equidistant points per year, creating a monthly time series. The time scale error in any given year is 1 to 2 months, due to interannual differences in the exact timing of peak SST. As such, AG covers the period 1982 to 2016 while EG covers 2006 to 2016. Application of lagged correlations to monthly/seasonal climate indices (Fig. 6) revealed the highest correlations at zero lag, suggesting that our linearly interpolated age model did not obfuscate seasonal trends in geochemical proxy data, despite the 1–2-month uncertainty in identifying annual anchor points.

### δ^18^O_sw_ reconstruction

For the calculation of δ^18^O_sw_ the method of Ren *et al*.^44^ was followed, with the assumption that coral Sr/Ca is solely a function of SST and that coral δ^18^O is a function of both SST and oxygen isotopic composition of the seawater. The method uses instantaneous changes of both variables instead of looking at the absolute values. Effects of seawater isotopy on coral δ^18^O are separated from thermal effects by breaking the instantaneous changes of coral δ^18^O into separate contributions by instantaneous SST and δ^18^O_sw_ changes, respectively. This is possible due to paired measurements of Sr/Ca and δ^18^O on the coral. In these calculations, we used a slope of −0.2‰ per 1 °C^82^ for δ^18^O_coral_ - SST, and for Sr/Ca-SST a slope of −0.06 mmol/mol °C^−1^ ^26^. Error estimation was performed by neglecting the error caused by non-climatic factors that may influence the proxies. The error σ_δ18Osw_ in this study is 0.103‰. It was calculated following the approach of Cahyarini *et al*.^38^ (Eq. 2), with σ_δc_ being the error of measured δ^18^O_coral_, σ_Sr/Ca_ being the error of measured Sr/Ca_coral_, and γ_1_ and β_1_ being the slopes of the linear regression of δ^18^O versus SST and Sr/Ca versus SST, respectively.

2 $${\sigma }_{\delta sw}^{2}={\sigma }_{\delta c}^{2}+{(\frac{{\gamma }_{1}}{{\beta }_{1}})}^{2}\times {\sigma }_{Sr/Ca}^{2}$$

Anomalies of δ^18^O_sw_ from all coral records and gridded SSS^65^ for the study area were calculated relative to the 2006 to 2015 mean using the KNMI Climate Explorer^80^.

### Historical climate data and climate indices

Local mean air temperature data from the Miri airport station (WMO station No. 964490, 4.4°N, 114.0°E, elevation: 51 m a.s.l.) were obtained from the Global Historical Climatology Network-Monthly (GHCN-M version 3) temperature quality-controlled dataset^83^. Data were provided by the National Centers for Environmental Information (NCEI) of the U.S. National Oceanic and Atmospheric Administration (NOAA), downloaded from KNMI Climate Explorer^80^. Local monthly precipitation data from the Miri airport station (WMO station No. 964490, 4.4°N, 114.0 °E, elevation: 51 m a.s.l) were obtained from the Global Historical Climatology Network Monthly (GHCN-M version 2) quality-controlled dataset, which contains temperature, precipitation, and pressure data^84^. Data were provided by NOAA’s NCEI, downloaded from KNMI Climate Explorer^80^.

River discharge data for the Baram River catchment were provided by the Department of Irrigation and Drainage (DID) in Malaysia^15^. The nearest station to the Baram outflow north of Miri was Marudi station (4.10°N, 114.18°E), which provided continuous data coverage between 1989 and 2015.

The NOAA 0.25° daily Optimum Interpolation Sea Surface Temperature version 2 (OISSTv2) dataset was used^46,47^. The dataset combines observations from ships, buoys and satellites (infrared satellite data from the Advanced Very High Resolution Radiometer AVHRR), with data gaps filled by interpolation. Daily SST data was available at a 0.25° grid resolution. The grid used in this study was 4.24–4.5°N, 113.75–114.00°E. Data were provided by NOAA’s NCEI, downloaded from KNMI Climate Explorer^80^.

EN4.2.0 SSS was obtained from the EN4 quality controlled subsurface ocean temperature and salinity compiled dataset, which is based on data of all instruments capable of profiling the water column^66^. Observations were interpolated and available in a grid resolution of 1°. The grid used in this study was 3.5–4.5°N, 113.5–114.5°E, but only surface salinity data were used. Data were provided by Met Office Hadley Centre, downloaded from KNMI Climate Explorer^80^.

ENSO indices Niño3.4 and Niño4 used in this study were based on OISSTv2^46^. Niño3.4 includes SST anomalies in the 5°N–5°S and 170°W–120°W region, Niño4 in the 5°N–5°S and 160° E–150°W region^51^. For both indices, anomalies were calculated relative to a 1981–2010 base period. The Niño 3.4 region represents the core region of ENSO. ENSO events were defined when 5-month running means of SST anomalies in the Niño 3.4 region exceeded a threshold of ±0.4 °C for a period of six months or more^85^.

## Supplementary information

Supplementary information.

## Data Availability

The coral proxy data from this publication will be archived after publication with the public NOAA WDC data portal at https://www.ncdc.noaa.gov/data-access/paleoclimatology-data/datasets.

## References

[1] Buddemeier, R.W., Kleypas, J. & Aronson, R. Coral reefs and Global climate change: Potential Contributions of Climate Change to Stresses on Coral Reef Ecosystems. Pew Center on Global Climate Change, Virginia, USA, 56 pp. (2004).

[2] Burke, L., Reytar, K., Spalding, M. & Perry, A. Reefs at Risk Revisited. World Resources Institute, Washington, D. C. (2011).

[3] Pilcher, N. & Cabanban, A. The status of coral reefs in Eastern Malaysia. Australian Institute of Marine Schience, Townsville, Australia (2000).

[4] Tun, K. *et al*. Status of coral reefs in Southeast Asia, in: Wilkinson, C. (Ed.), Status of coral reefs of the world. Global Coral Reef Monitoring Network and Reef and Rainforest Research Centre, Townsville, Australia, pp. 131–144 (2008).

[5] Wilkinson, C. Status of coral reefs of the world: 2008. Global Coral Reef Monitoring Network and Reef and Rainforest Research Centre, Townsville, Australia.

[6] Wilkinson, C. C., DeVantier, L. L., Talau-McMaanus, L. L. & Souter, D. D. South China Sea; GIWA Regional Assessment 54. UNEP and University of Kalmar, Sweden (2005).

[7] Gaveau DLA (2016). Rapid conversions and avoided deforestation: examining four decades of industrial plantation expansion in Borneo. Scientific Reports.

[8] Gaveau DLA (2014). Four Decades of Forest Persistence, Clearance and Logging on Borneo. Plos One.

[9] Hansen MC (2013). High-Resolution Global Maps of 21st-Century Forest Cover Change. Science.

[10] Hansen MC (2008). Humid tropical forest clearing from 2000 to 2005 quantified by using multitemporal and multiresolution remotely sensed data. Proceedings of the National Academy of Sciences.

[11] Fabricius KE (2005). Effects of terrestrial runoff on the ecology of corals and coral reefs: review and synthesis. Marine Pollution Bulletin.

[12] MacNeil MA (2019). Water quality mediates resilience on the Great Barrier Reef. Nature Ecology & Evolution.

[13] Stephens M, Rose J (2005). Modern stable isotopic (δ18O, δ2H, δ13C) variation in terrestrial, fluvial, estuarine and marine waters from north-central Sarawak, Malaysian Borneo. Earth Surface Processes and Landforms.

[14] Tangang FT (2012). Climate change and variability over Malaysia: Gaps in science and research information. Sains Malaysiana.

[15] Sa’adi Z, Shahid S, Ismail T, Chung E-S, Wang X-J (2017). Distributional changes in rainfall and river flow in Sarawak, Malaysia. Asia-Pacific Journal of Atmospheric Sciences.

[16] Salahuddin A, Curtis S (2011). Climate extremes in Malaysia and the equatorial South China Sea. Global and Planetary Change.

[17] Sa’adi Z, Shahid S, Ismail T, Chung E-S, Wang X-J (2017). Trends analysis of rainfall and rainfall extremes in Sarawak, Malaysia using modified Mann–Kendall test. Meteorology and Atmospheric Physics.

[18] Fuller DO, Murphy K (2006). The Enso-Fire Dynamic in Insular Southeast Asia. Climatic Change.

[19] Tangang FT, Juneng L (2004). Mechanisms of Malaysian Rainfall Anomalies. Journal of Climate.

[20] Chen C-C, Lin H-W, Yu J-Y, Lo M-H (2016). The 2015 Borneo fires: What have we learned from the 1997 and 2006 El Niños?. Environmental Research Letters.

[21] Tangang F (2017). Characteristics of precipitation extremes in Malaysia associated with El Niño and La Niña events. International Journal of Climatology.

[22] Sloan S, Locatelli B, Wooster MJ, Gaveau DLA (2017). Fire activity in Borneo driven by industrial land conversion and drought during El Nino periods, 1982–2010. Global Environ. Change.

[23] Krishnamurthy L, Krishnamurthy V (2014). Influence of PDO on South Asian summer monsoon and monsoon–ENSO relation. Climate Dynamics.

[24] Tierney JE (2015). Tropical sea-surface temperatures for the past four centuries reconstructed from coral archives. Paleoceanography.

[25] Saha N, Webb GE, Zhao J-X (2016). Coral skeletal geochemistry as a monitor of inshore water quality. Science of the Total Environment.

[26] Corrège T (2006). Sea surface temperature and salinity reconstruction from coral geochemical tracers. Palaeogeography, Palaeoclimatology, Palaeoecology.

[27] de Villiers S, Nelson BK, Chivas AR (1995). Biological Controls on Coral Sr/Ca and δ18O Reconstructions of Sea Surface Temperatures. Science.

[28] DeLong KL, Quinn TM, Taylor FW, Shen C-C, Lin K (2013). Improving coral-base paleoclimate reconstructions by replicating 350 years of coral Sr/Ca variations. Palaeogeography, Palaeoclimatology, Palaeoecology.

[29] Gagan MK, Dunbar GB, Suzuki A (2012). The effect of skeletal mass accumulation in Porites on coral Sr/Ca and δ18O paleothermometry. Paleoceanography.

[30] Pfeiffer M, Dullo W-C, Zinke J, Garbe-Schönberg D (2009). Three monthly coral Sr/Ca records from the Chagos Archipelago covering the period of 1950–1995 A.D.: reproducibility and implications for quantitative reconstructions of sea surface temperature variations. International Journal of Earth Sciences.

[31] DeCarlo Thomas M., Gaetani Glenn A., Cohen Anne L., Foster Gavin L., Alpert Alice E., Stewart Joseph A. (2016). Coral Sr‐U thermometry. Paleoceanography.

[32] Epstein S, Buchsbaum R, Lowenstam HA, Urey HC (1953). Revised Carbonate-Water Isotopic Temperature Scale. GSA Bulletin.

[33] Urey HC, Epstein S, McKinney C, McCrea J (1948). Method for measurement of paleotemperatures. Bulletin of the Geological Society of America.

[34] Urey HC, Epstein S, McKinney CR (1951). Measurements of palaeotemperatures and temperatures of the Upper Cretaceous of England. GSA Bulletin.

[35] Weber JN, Woodhead PMJ (1972). Temperature dependence of oxygen‐18 concentration in reef coral carbonates. Journal of Geophysical Research.

[36] Cobb KM, Charles CD, Hunter DE (2001). A central tropical Pacific coral demonstrates Pacific, Indian, and Atlantic decadal climate connections. Geophysical Research Letters.

[37] Zinke J, Dullo WC, Heiss GA, Eisenhauer A (2004). ENSO and Indian Ocean subtropical dipole variability is recorded in a coral record off southwest Madagascar for the period 1659 to 1995. Earth and Planetary Science Letters.

[38] Cahyarini SY, Pfeiffer M, Timm O, Dullo W-C, Schönberg DG (2008). Reconstructing seawater δ18O from paired coral δ18O and Sr/Ca ratios: Methods, error analysis and problems, with examples from Tahiti (French Polynesia) and Timor (Indonesia). Geochimica et Cosmochimica Acta.

[39] Nurhati IS, Cobb KM, Di Lorenzo E (2011). Decadal-Scale SST and Salinity Variations in the Central Tropical Pacific: Signatures of Natural and Anthropogenic Climate Change. Journal of Climate.

[40] Lough JM (2010). Climate records from corals. Wiley Interdisciplinary Reviews. Climate Change.

[41] Bolton A (2014). Paired Porites coral Sr/Ca and δ18O from the western South China Sea: Proxy calibration of sea surface temperature and precipitation. Palaeogeography, Palaeoclimatology, Palaeoecology.

[42] Gagan MK (1998). Temperature and Surface-Ocean Water Balance of the Mid-Holocene Tropical Western. Pacific. Science.

[43] McCulloch MT, Gagan MK, Mortimer GE, Chivas AR, Isdale PJ (1994). A high-resolution Sr/Ca and δ18O coral record from the Great Barrier Reef, Australia, and the 1982–1983 El Niño. Geochimica et Cosmochimica Acta.

[44] Ren L, Linsley BK, Wellington GM, Schrag DP, Hoegh-Guldberg O (2003). Deconvolving the δ18O seawater component from subseasonal coral δ18O and Sr/Ca at Rarotonga in the southwestern subtropical Pacific for the period 1726 to 1997. Geochimica et Cosmochimica Acta.

[45] Browne N, Braoun C, McIlwain J, Nagarajan R, Zinke J (2019). Borneo coral reefs subject to high sediment loads show evidence of resilience to various environmental stressors. PeerJ.

[46] Reynolds RW (2007). Daily High-Resolution-Blended Analyses for Sea Surface Temperature. Journal of Climate.

[47] Banzon V, Smith TM, Chin C, Liu C, Hankins W (2016). A long-term record of blended satellite and *in situ* sea-surface temperature for climate monitoring, modeling and environmental studies. Earth System Science Data.

[48] Sun Y (2004). Strontium contents of a Porites coral from Xisha Island, South China Sea: A proxy for sea-surface temperature of the 20th century. Paleoceanography.

[49] Wei G, Sun M, Li X, Nie B (2000). Mg/Ca, Sr/Ca and U/Ca ratios of a porites coral from Sanya Bay, Hainan Island, South China Sea and their relationships to sea surface temperature. Palaeogeography, Palaeoclimatology, Palaeoecology.

[50] Yu K-F, Zhao J-X, Wei G-J, Cheng X-R, Wang P-X (2005). Mid–late Holocene monsoon climate retrieved from seasonal Sr/Ca and δ18O records of Porites lutea corals at Leizhou Peninsula, northern coast of South China Sea. Global and Planetary Change.

[51] Kaplan A (1998). Analyses of global sea surface temperature 1856–1991. Journal of Geophysical Research: Oceans.

[52] Mantua NJ, Hare SR, Zhang Y, Wallace JM, Francis RC (1997). A Pacific interdecadal climate oscillation with impacts on salmon production. Bull. Amer. Meteor. Soc..

[53] Yu K (2001). The high-resolution climate recorded in the δ18O of Porites lutea from the Nansha Islands of China. Chinese Science Bulletin.

[54] Heron SF, Maynard JA, van Hooidonk R, Eakin CMW (2016). Trends and Bleaching Stress of the World’s Coral Reefs 1985–2012. Scientific Reports.

[55] Bao B, Ren G (2014). Climatological characteristics and long-term change of SST over the marginal seas of China. Continental Shelf Research.

[56] He S (2013). Reduction of the East Asian winter monsoon interannual variability after the mid-1980s and possible cause. Chinese Science Bulletin.

[57] Wang, P. & Li, Q. Oceanographical and Geological Background, in: Wang, P. & Li, Q. (Eds.), T*he South China Sea: Paleoceanography and Sedimentology*. Springer Netherlands, Dordrecht, pp. 25–73 (2009).

[58] Juneng L, Tangang FT (2005). Evolution of ENSO-related rainfall anomalies in Southeast Asia region and its relationship with atmosphere-ocean variations in Indo-Pacific sector. Climate Dynamics.

[59] Tan W, Wang X, Wang W, Wang C, Zuo J (2016). Different Responses of Sea Surface Temperature in the South China Sea to Various El Niño Events during Boreal Autumn. Journal of Climate.

[60] Shi Q (2012). Two centuries-long records of skeletal calcification in massive Porites colonies from Meiji Reef in the southern South China Sea and its responses to atmospheric CO_2_ and seawater temperature. Science China Earth Sciences.

[61] Power S, Casey T, Folland C, Colman A, Mehta V (1999). Interdecadal modulation of the impact of ENSO on Australia. Clim. Dynam..

[62] Moerman JW (2013). Diurnal to interannual rainfall δ18O variations in northern Borneo driven by regional hydrology. Earth and Planetary Sci. Lett..

[63] Kurita N (2018). Interpretation of El Niño–Southern Oscillation-related precipitation anomalies in north-western Borneo using isotopic tracers. Hydrological Processes.

[64] Fairbanks RG (1997). Evaluating climate indices and their geochemical proxies measured in corals. Coral Reefs.

[65] Rohling, E.J. Oxygen Isotopte Composition of Seawater, in: Elias, S. A. (Ed.), *The Encyclopedia of Quaternary Science*. Elsevier, Amsterdam, pp. 951–922 (2013).

[66] Good SA, Martin MJ, Rayner NA (2013). EN4: Quality controlled ocean temperature and salinity profiles and monthly objective analyses with uncertainty estimates. Journal of Geophysical Research: Oceans.

[67] Zeng L, Chassignet EP, Schmitt RW, Xu X, Wang D (2018). Salinification in the South China Sea since Late 2012: A Reversal of the Freshening Since the 1990s. Geophysical Research Letters.

[68] Deng W (2013). Variations in the Pacific Decadal Oscillation since 1853 in a coral record from the northern South China Sea. Journal of Geophysical Research: Oceans.

[69] Cobb KM, Adkins JF, Partin JW, Clark B (2007). Regional-scale climate influences on temporal variations of rainwater and cave dripwater oxygen isotopes in northern Borneo. Earth and Planetary Science Letters.

[70] Chen S (2016). A high-resolution speleothem record of western equatorial Pacific rainfall: Implications for Holocene ENSO evolution. Earth and Planetary Science Letters.

[71] Nakagawa M (2000). Impact of severe drought associated with the 1997–1998 El Niño in a tropical forest in Sarawak. Journal of Tropical Ecology.

[72] Neale R, Slingo J (2003). The Maritime Continent and Its Role in the Global Climate: A GCM Study. Journal of Climate.

[73] Lau N-C, Nath MJ (2003). Atmosphere–Ocean Variations in the Indo-Pacific Sector during ENSO Episodes. Journal of Climate.

[74] Ropelewski CF, Halpert MS (1987). Global and Regional Scale Precipitation Patterns Associated with the El Niño/Southern Oscillation. Monthly Weather Review.

[75] Nagtegaal R (2012). Spectral luminescence and geochemistry of coral aragonite: Effects of whole-core treatment. Chemical Geology.

[76] Schrag DP (1999). Rapid analysis of high-precision Sr/Ca ratios in corals and other marine carbonates. Paleoceanography.

[77] de Villiers S, Greaves M, Elderfield H (2002). An intensity ratio calibration method for the accurate determination of Mg/Ca and Sr/Ca of marine carbonates by ICP-AES. Geochemistry, Geophysics, Geosystems.

[78] Hathorne EC (2013). Interlaboratory study for coral Sr/Ca and other element/Ca ratio measurements. Geochemistry, Geophysics, Geosystems.

[79] Solow AR, Huppert A (2004). A potential bias in coral reconstruction of sea surface temperature. Geophysical Research Letters.

[80] Trouet V, Van Oldenborgh GJ (2013). KNMI Climate Explorer: A Web-Based Research Tool for High-Resolution Paleoclimatology. Tree-Ring Research.

[81] Paillard D, Labeyrie L, Yiou P (1996). Macintosh program performs time-series analysis. EOS Trans. Am. Geophys. Union.

[82] Juillet-Leclerc A, Schmidt G (2001). A calibration of the oxygen isotope paleothermometer of coral aragonite from porites. Geophysical Research Letters.

[83] Lawrimore JH (2011). An overview of the Global Historical Climatology Network monthly mean temperature data set, version 3. Journal of Geophysical Research: Atmospheres.

[84] Peterson TC, Vose RS (1997). An Overview of the Global Historical Climatology Network Temperature Database. Bulletin of the American Meteorological Society.

[85] Trenberth KE (1997). The Definition of El Niño. Bulletin of the American Meteorological Society.

[86] Schlitzer, R. Ocean Data View 5.0.0, https://odv.awi.de (2018).

[87] Antonov, J. I. *et al*. World Ocean Atlas 2009, Volume 2: Salinity, in: Levitus, S. (Ed.), NOAA Atlas NESDIS 69. U.S. Government Printing Office, Washington, D.C., p. 184 (2010).

[88] Locarnini, R. A. *et al*. World Ocean Atlas 2009, Volume 1: Temperature, in: Levitus, S. (Ed.), NOAA Atlas NESDIS 68. U.S. Government Printing Office, Washington, D.C., p. 184 (2010).

[89] QGIS Development Team. QGIS Geographic Information System. Open Source Geospatial Foundation Project, http://qgis.osgeo.org (2019).

