# Supplementary material for: Corals reveal ENSO-driven synchrony of climate impacts on both terrestrial and marine ecosystems in northern Borneo
Source: Sci Rep. 2020 Feb 28;10:3678. doi: 10.1038/s41598-020-60525-1 (PMC7048730; doi:10.1038/s41598-020-60525-1)
Supplement: Supplementary file 1 — Supplementary information. [file 41598_2020_60525_MOESM1_ESM.pdf]

Corals reveal ENSO-driven synchrony of climate impacts on both terrestrial and marine ecosystems in northern Borneo

Krawczyk, Hedwig, Zinke, Jens, Browne, Nicola, Struck, Ulrich, McIlwain, Jennifer, O'Leary, Michael, Garbe-Schönberg, Dieter

Supplementary Information

Details on study area and climatic setting

The AG and EG reefs are located in the southern South China Sea (SCS), the largest semi-enclosed marginal sea in SEA. It extends over an area of 186,930 ha (see **Fig. 2** main text) including shallow inshore reefs (5-15 m depth) and extensive submerged offshore reefs (~30 m depth) (Browne et al., 2019). Sediment influx from nearby rivers is a major concern for the health status of the reefs (Pilcher and Cabanban, 2000; Browne et al., 2019). The two main runoff sources into the MSCRNP are the Miri estuary and the Baram River (see **Fig. 2** main text). The Baram River has a catchment area of approximately 22,800 km² and its sediment discharge is approximately 2.4 x 10^{10} kg (Lambiase et al., 2002; Staub & Esterle, 1994).
Sarawak's tropical equatorial climate is characterized by low seasonal temperature seasonality and seasonally varying rainfall (Hua et al., 2013; Sa'adi et al., 2017a). Miri's airport is the closest weather station to our study sites which is included in the Global Historical Climatology Network-Monthly data set (GHCN-M) (Lawrimore et al., 2011). The Miri station data consists of a patchy monthly time series of air temperature and precipitation, continuous since 1992. Highest precipitation was found between October and January while it is lowest between May and September. However, precipitation in Borneo is more evenly distributed across the annual cycle and less monsoonal (Salahuddin and Curtis, 2011). Intra-monsoon rainfall occurs in April and October in any given year, with the latter often leading to wetter conditions and lower salinities in the southern SCS (Sa'adi et al., 2017). Local monthly air temperatures in Miri range between 26.4 and 27.8°C, and monthly precipitation ranges between 153 and 346 mm (**Fig. S1**).
SSTs from satellite data (AVHRR OISSTv2; Banzon et al., 2016) at the northern Borneo coast are highest from April to June (AMJ; 30.8ºC) and drop to 25.9 ºC from January to March (JFM) (Locarnini et al., 2010; Banzon et al., 2016; Reynolds et al., 2007). A 10-month *in situ* SST dataset (measured with HOBO Pro V2 loggers, Australia) between September 2016 and May 2017 from two reefs sites in the MSCRNP largely agrees with the timing of warm and cold seasons in AVHRR-OISSTv2 (Browne et al., 2019; **Fig. S2**). However, *in situ* SST was on average significantly warmer than AVHRR-OISSTv2 with the exception of March 2017 (Browne et al., 2019). Satellite SST data can be biased in nearshore areas, for example due to high cloud coverage and "land bleed" (i.e. mixing of land temperature with SST) (Zhang et al., 2004; Brewin et al., 2017; Pearce et al., 2006; Smit et al., 2013). Surface salinity from the EN4 dataset ranged between 32.05 and 34.07 psu, with a mean of 33.10 psu from 1982 to 2016 with lowest salinities (32 psu) between October and January (ONDJ; **see Fig. 1 in main text**), which is also the season of highest rainfall (Antonov et al., 2010; **Fig. S1**).

Variability in SST reconstructions, local and regional comparison

The comparison between SST reconstructions from EG and AG reefs showed good agreement with both absolute instrumental SSTs and anomalies for most years with discrepancies in annual amplitudes, especially before 2006. The lack of SST variation between reefs in our cores, despite differences in the depth and distance from shore, was verified by temperature

loggers deployed at both reefs from September 2016 to May 2017 (Browne et al., 2019). These *in situ* measurements showed only small variations between the reefs, with a maximum difference of 0.48 °C in March (**Fig. S2**). In contrast, the comparison of *in situ* SST and AVHRR-OISSTv2 showed temperature discrepancies of almost 1°C in some months. This suggests that AVHRR-OISSTv2 might not be an optimal choice as calibration dataset. Ideally, local *in situ* SST should be used to obtain most accurate temperature reconstructions, as for example shown in a comparative study by Corrège (2006) on a *Porites* coral from New Caledonia. Since only short *in situ* SST was available for the study area, the calibration of this study was based on the 0.25° gridded AVHRR-OISSTv2 (Banzon et al., 2016). The calibration slopes with AVHRR-OISSTv2 obtained in this study (-0.043 to -0.039 mmol/mol $°C^{-1}$) were on the higher end of various published slopes ranging between -0.04 mmol/mol $°C^{-1}$ and -0.084 mmol/mol $°C^{-1}$ (e.g. Corrège, 2006; Gagan et al., 2012 and references therein). However, slopes of other coral core studies in the northern SCS were similar (Sun et al., 2004; Wei et al., 2000; Yu et al., 2005).

The coral Sr/Ca-SST showed strikingly better agreement with the AVHRR-OISSTv2 from 2006, which might be associated with the change of used instruments. From 2006, the Operational U.S. Navy AVHRR data was available, which utilized several AVHRR instruments. The Pathfinder AVHHR was the only instrument used for OISSTv2 from 1985 to 2006 (Reynolds et al., 2007). AVHRR-OISSTv2 used *in situ* data from ships and buoys to minimize biases from the satellite measurements (Banzon et al., 2016). This kind of quality control depends on the availability of observational data, which is rather sparse in the study area. Despite these issues, the differences between AVHRR-OISSTv2 and coral-derived Sr/Ca-SST are within the error range of the reconstruction. Furthermore, a comparison of reconstructed absolute SST as well as SST anomalies with the gridded SST data indicates similar trends in the records.

References

Antonov, J.I. *et al*. World Ocean Atlas 2009, Volume 2: Salinity, in: Levitus, S. (Ed*.), NOAA Atlas NESDIS 69*. U.S. Government Printing Office, Washington, D.C., p. 184 (2010).

Banzon, V., Smith, T.M., Chin, C., Liu, C. and Hankins, W. A long-term record of blended satellite and in situ sea-surface temperature for climate monitoring, modeling and environmental studies. *Earth System Science Data* **8**, 165-176 (2016).

Beaulieu, C., Chen, J., Sarmiento, J. L. Change-point analysis as a tool to detect abrupt climate variations. Phil. Trans. Roy. Soc. A **370**, 1228-1249 (2012).

Brewin, R.J.W. *et al*. Evaluating operational AVHRR sea surface temperature data at the coastline using surfers. *Estuarine, Coastal and Shelf Science* **196**, 276-289 (2017).

Browne, N., Braoun, C., McIlwain, J., Nagarajan, R., Zinke, J. Borneo coral reefs subject to high sediment loads show evidence of resilience to various environmental stressors. *PeerJ* **7**, e7382, doi:10.7717/peerj.7382 (2019).

Corrège, T. Sea surface temperature and salinity reconstruction from coral geochemical tracers. *Palaeogeography, Palaeoclimatology, Palaeoecology* **232**, 408-428 (2006).

Gagan, M.K., Dunbar, G.B. and Suzuki, AThe effect of skeletal mass accumulation in Porites on coral Sr/Ca and $\delta^{18}O$ paleothermometry. *Paleoceanography* **27**, PA1203 (2012).

Hua, T.M., Hui, R.C.Y. and Husen, R. Trends of rainfall in Sarawak from 1999 to 2008. Proceedings of The International Conference on Social Science Research, Penang, Malaysia, pp. 261-269 (2013).

Lambiase, J.J., Rahim, A.A.b.A. and Peng, C.Y. Facies distribution and sedimentary processes on the modern Baram Delta: implications for the reservoir sandstones of NW Borneo. *Marine and Petroleum Geology* **19**, 69-78 (2002).

Lawrimore, J.H. *et al.* An overview of the Global Historical Climatology Network monthly mean temperature data set, version 3. *Journal of Geophysical Research: Atmospheres* **116**, D19921 (2011).

Locarnini, R.A. *et al.* World Ocean Atlas 2009, Volume 1: Temperature, in: Levitus, S. (Ed.), *NOAA Atlas NESDIS 68*. U.S. Government Printing Office, Washington, D.C., p. 184 (2010).
Pearce, A., Faskel, F. and Hyndes, G. Nearshore sea temperature variability off Rottnest Island (Western Australia) derived from satellite data. *International Journal of Remote Sensing* **27**, 2503-2518 (2006)

Peterson, T.C. and Vose, R.S. An Overview of the Global Historical Climatology Network Temperature Database. *Bulletin of the American Meteorological Society* **78**, 2837- 2850 (1997).

Pilcher, N. and Cabanban, A. The status of coral reefs in Eastern Malaysia. Australian Institute of Marine Schience, Townsville, Australia (2000).

R Core Team R: A language and environment for statistical computing. R Foundation for Statistical Computing, Vienna, Austria. URL http://www.R-project.org/ (2013).

Reynolds, R.W. *et al.* Daily High-Resolution-Blended Analyses for Sea Surface Temperature. *Journal of Climate* **20**, 5473-5496 (2007).

Sa'adi, Z., Shahid, S., Ismail, T., Chung, E.-S. and Wang, X.-J. Distributional changes in rainfall and river flow in Sarawak, Malaysia. Asia-Pacific *Journal of Atmospheric Sciences* **53**, 489-500 (2017a).

Salahuddin, A. and Curtis, S. Climate extremes in Malaysia and the equatorial South China Sea. *Global and Planetary Change* **78**, 83-91 (2011).

Smit, A.J., Roberts, M., Anderson, R.J., Dufois, F., Dudley, S.F.J., Bornman, T.G., Olbers, J. and Bolton, J.J. A Coastal Seawater Temperature Dataset for Biogeographical Studies: Large Biases between In Situ and Remotely-Sensed Data Sets around the Coast of South Africa. *PLOS ONE* **8**, e81944 (2013).

Staub, J.R. and Esterle, J.S. Peat-accumulating depositional systems of Sarawak, East Malaysia. *Sedimentary Geology* **89**, 91-106 (1994).

Sun, Y. *et al.* Strontium contents of a *Porites* coral from Xisha Island, South China Sea: A proxy for sea-surface temperature of the 20th century. *Paleoceanography* **19**, PA2004 (2004).

Trouet, V. and Van Oldenborgh, G.J. KNMI Climate Explorer: A Web-Based Research Tool for High-Resolution Paleoclimatology. *Tree-Ring Research* **69**, 3-13 (2013).

Wei, G., Sun, M., Li, X. and Nie, B. Mg/Ca, Sr/Ca and U/Ca ratios of a porites coral from Sanya Bay, Hainan Island, South China Sea and their relationships to sea surface temperature. *Palaeogeography, Palaeoclimatology, Palaeoecology* **162**, 59-74 (2000).

Yu, K.-F., Zhao, J.-X., Wei, G.-J., Cheng, X.-R. and Wang, P.-X. Mid–late Holocene monsoon climate retrieved from seasonal Sr/Ca and $\delta^{18}O$ records of Porites lutea corals at Leizhou Peninsula, northern coast of South China Sea. *Global and Planetary Change* **47**, 301-316 (2005).

Zhang, H.M., Reynolds, R.W. and Smith, T.M. Bias characteristics in the AVHRR sea surface temperature. *Geophysical Research Letters* **31** (2004).

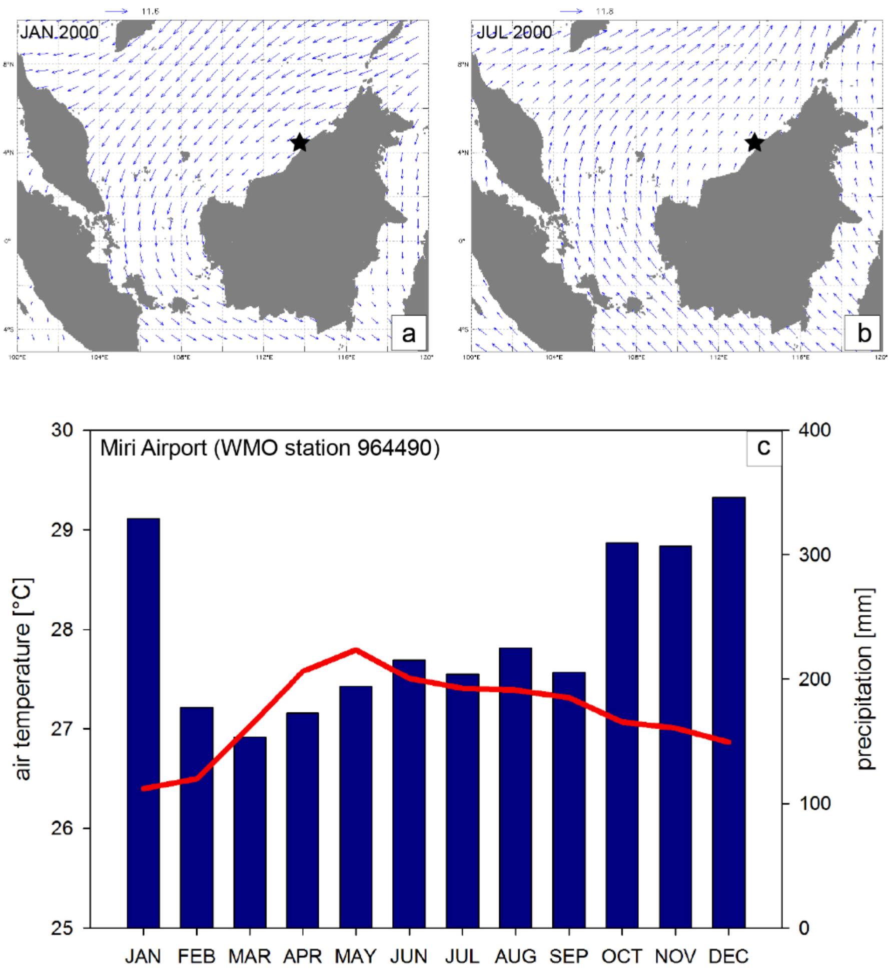

Figure S1 – Monthly wind speed [m/s] and direction for boreal winter (a) and summer (b). Study area is marked with black star. Figures made with MY NASA DATA Live Access Server-Advanced. Data: Monthly Ocean Wind Speed Vectors (NOAA NOMADS). c) Mean monthly air temperature (Lawrimore et al., 2011) and precipitation (Peterson and Vose, 1997) measured at Miri airport weather station, based on 1997-2017 averages. (WMO station No. 964490).

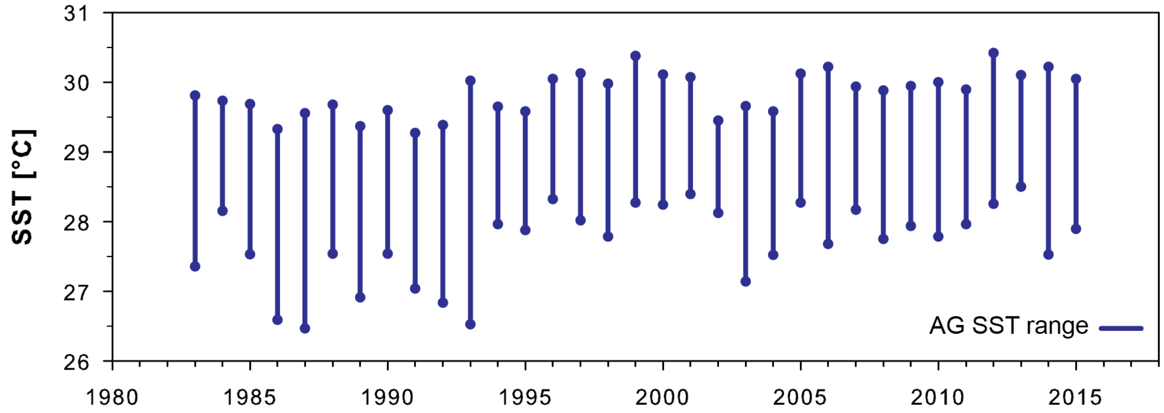

Figure S2 – AG Sr/Ca-SST time series for annual ranges. Note the higher winter SSTs post 1993. The mean seasonal range pre-1993 (2.45±0.27ºC; N= 11) and post-1993 (2±0.11ºC; N= 21) differ significantly according to a two-tailed t-test (t= 2.59, p= 0.02).

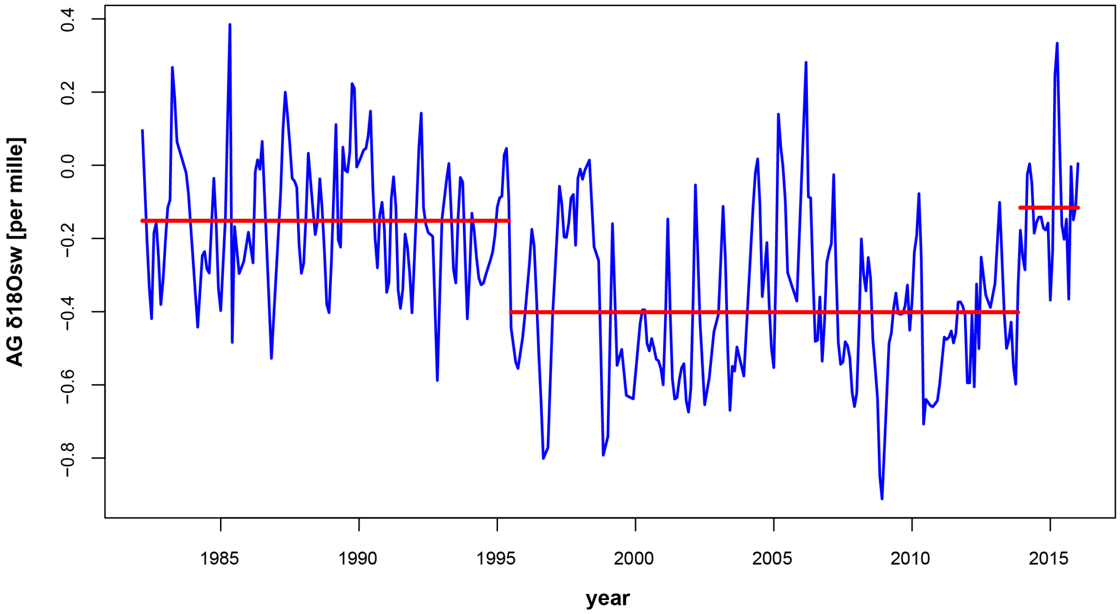

Figure S3 – Change-point analysis in mean and variance of coral AG $\delta^{18}O_{sw}$ time series following the methods described in Beaulieu et al. (2012). Note the change-point in 1995 from positive to negative $\delta^{18}O_{sw}$ anomalies followed by positive anomalies post-2013. The red line marks the mean of each subperiod that showed statistically significant differences. Analyis was done in R version 3.4.3 (R core team, 2013).

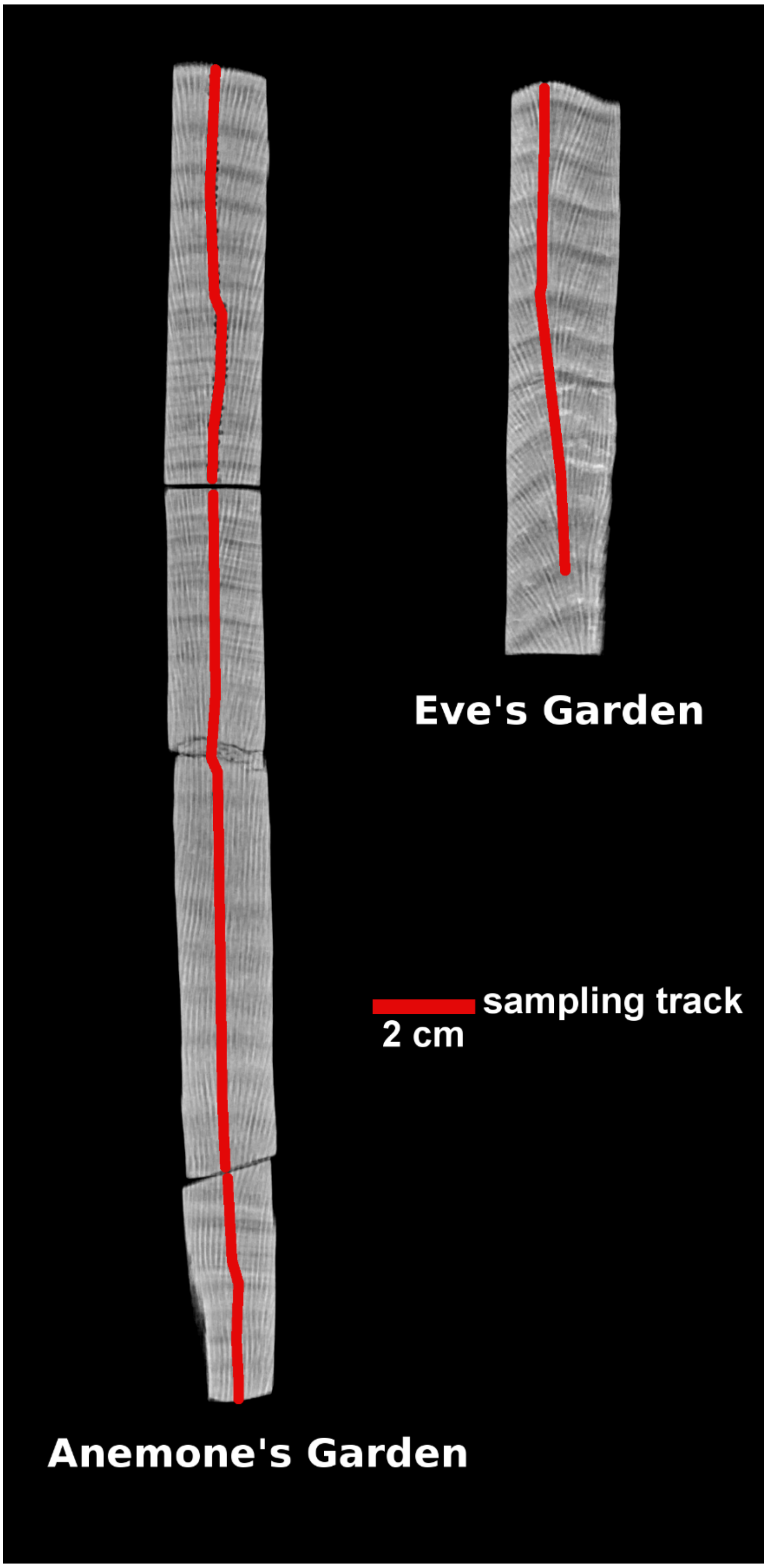

Figure S4 - X-ray images of coral cores with sampling tracks marked in red.

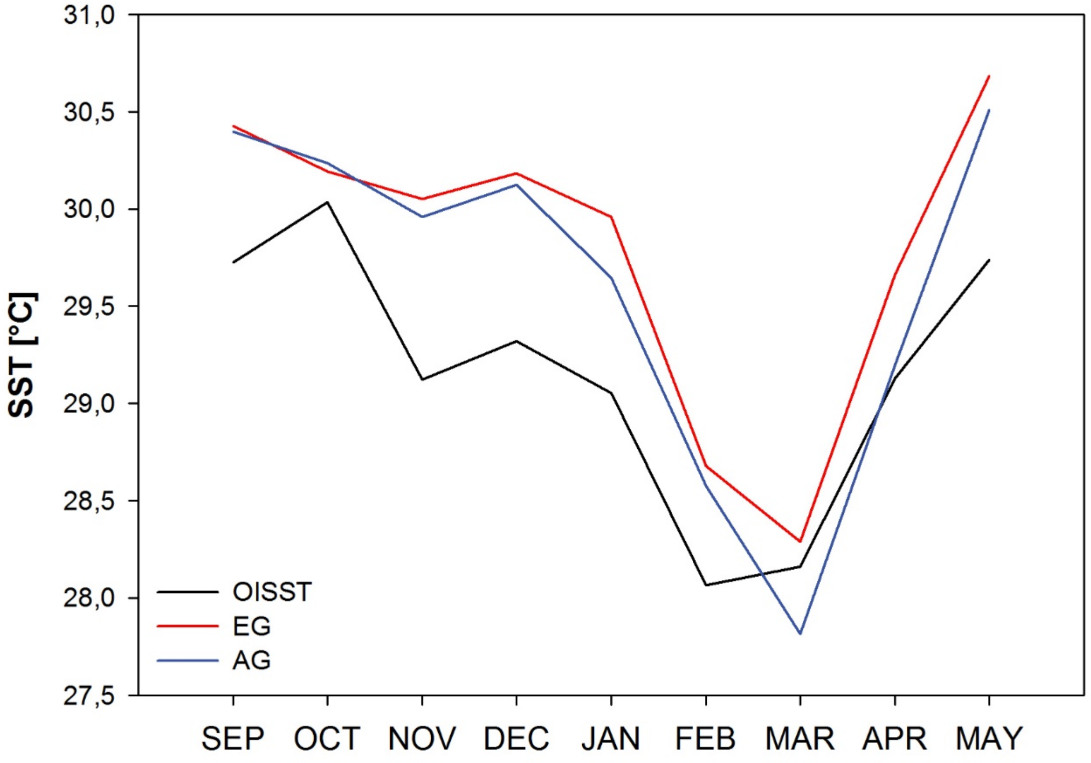

Figure S5 - Monthly mean *in situ* SST between September 2016 and May 2017 based on bi-hourly temperature logger data from AG and EG reefs compared to OISSTv2 (Reynolds et al., 2007).

Table S1 - Descriptive statistics of all geochemical proxies for cores AG and EG. Statistics shown for period 2006 to 2016 where AG and EG overlap and full length of core AG 1982 to 2016. AG descriptive statistics also indicated for data between 1982 and 2006.

	**mean**	**median**	**std. dev.**	**min.**	**max.**
**Sr/Ca [mmol/mol]**					
**AG**					
1982-2016	8.82	8.81	0.06	8.73	9.00
1982-2006	8.83	8.82	0.06	8.73	9.00
2006-2016	8.80	8.78	0.05	8.73	8.93
**EG**					
2006-2016	8.80	8.79	0.04	8.72	8.93
**$\delta^{18}O$ [‰]**					
**AG**					
1982-2016	-6.60	-6.63	0.35	-7.37	-5.71
1982-2006	-6.54	-6.57	0.35	-7.35	-5.71
2006-2016	-6.74	-6.80	0.33	-7.37	-5.72
**EG**					
2006-2016	-6.48	-6.47	0.32	-7.41	-5.68
**$\delta^{18}O_{sw}$ [‰]**					
**AG**					
1982-2016	-0.29	-0.28	0.23	-0.91	0.38
1982-2006	-0.25	-0.24	0.23	-0.80	0.38
2006-2016	-0.36	-0.37	0.22	-0.91	0.33
**EG**					
2006-2016	-0.38	-0.35	0.26	-1.19	0.18

Table S2 - Summarized SST values of each site and of instrumental SST (Reynolds et al., 2007). Shown for each sampling period, and additionally for AG for overlapping period.

	**AG**	**NOAA OISSTv2**	**AG**	**EG**	**NOAA OISSTv2**
**time**	1982-2016	1982-2016	2006-2016	2006-2016	2006-2016
**min [°C]**	26.46	25.94	27.52	26.93	26.55
**max [°C]**	30.42	30.81	30.42	30.45	30.42
**mean [°C]**	29.05	29.05	29.36	29.16	29.15
**median [°C]**	29.24	29.34	29.67	29.33	29.44

Table S3 - Correlation coefficients of Miri AVHRR-OISSTv2 and Sr/Ca ratios of AG and EG with Niño3.4, Niño4 and PDO indices averaged over 3 months. All data detrended with 95% confidence intervals indicated in last column. P-values (p) and numbers of years (n) for correlations indicated. Correlations made with KNMI Climate Explorer (Trouet and Van Oldenborgh, 2013).

**averaged over 3 months**						
		**months**	**r**	**p**	**n**	**95% CI**
**AVHRR-OISSTv2**	NINO3.4	Feb-Apr	-0.17	0.325	34	-0.40; 0.12
	NINO4	Feb-Apr	-0.35	0.048	34	-0.57; -0.03
	PDO	Feb-Apr	-0.41	0.015	34	-0.61; -0.01
**AG Sr/Ca**	NINO3.4	Dec-Feb	0.32	0.065	34	0.02; 0.52
	NINO4	Feb-Apr	0.38	0.025	34	0.03; 0.60
	PDO	Apr-Jun	0.27	0.128	34	0.06; 0.52
**EG Sr/Ca**	NINO3.4	Aug-Oct	0.45	0.194	10	-0.71; 0.72
	NINO4	Jul-Sep	0.48	0.163	10	-0.73; 0.80
	PDO	Feb-Apr	0.57	0.086	10	-0.13; 0.91

Table S4 - Correlation coefficients and p-values of seasonal coral-derived (detrended) $\delta^{18}O_{sw}$ with salinity (EN4 SSS; Good et al., 2013), Miri station precipitation (Peterson and Vose, 1997) and Marudi station river discharge (Sa'adi et al., 2017).

	**Month**	**time**	**r**	**p**
**EN4 SSS vs. $\delta^{18}O_{sw}$**				
**AG**	**Feb-Apr**	1982-2016	0.59	<0.001
**EG**	**Jan-Mar**	2006-2016	0.75	0.020
**Local precipitation Miri station vs. $\delta^{18}O_{sw}$ and SSS**				
**AG**	**Feb-Apr**	1982-2016	-0.75	<0.001
**EG**	**Jan-Mar**	2006-2016	-0.92	<0.001
**SSS**	**Feb-Apr**	1991-2016	-0.53	0.006
**Marudi river discharge vs. $\delta^{18}O_{sw}$, EN4 SSS and precipitation**				
**AG**	**Jan-Mar**	1989-2016	-0.54	0.004
**EG**	**Jan-Mar**	2006-2016	-0.76	0.017
**SSS**	**Feb-Apr**	1989-2016	-0.35	0.070
**Prcp.**	**Feb-Apr**	1991-2016	0.84	<0.001
